# Insights from deconvolution of cell subtype proportions enhance the interpretation of functional genomic data

**DOI:** 10.1371/journal.pone.0215987

**Published:** 2019-04-25

**Authors:** Yu Kong, Deepa Rastogi, Cathal Seoighe, John M. Greally, Masako Suzuki

**Affiliations:** 1 Department of Genetics and Center for Epigenomics, Albert Einstein College of Medicine, Bronx, New York, United States of America; 2 Department of Pediatrics, Albert Einstein College of Medicine, Bronx, New York, United States of America; 3 School of Mathematics, Statistics and Applied Mathematics, National University of Ireland Galway, University Road, Galway, Ireland; Centro Cardiologico Monzino, ITALY

## Abstract

Cell subtype proportion variability between samples contributes significantly to the variation of functional genomic properties such as gene expression or DNA methylation. Although the impact of the variation of cell subtype composition on measured genomic quantities is recognized, and some innovative tools have been developed for the analysis of heterogeneous samples, most functional genomics studies using samples with mixed cell types still ignore the influence of cell subtype proportion variation, or just deal with it as a nuisance variable to be eliminated. Here we demonstrate how harvesting information about cell subtype proportions from functional genomics data can provide insights into cellular changes associated with phenotypes. We focused on two types of mixed cell populations, human blood and mouse kidney. Cell type prediction is well developed in the former, but not currently in the latter. Estimating the cellular repertoire is easier when a reference dataset from purified samples of all cell types in the tissue is available, as is the case for blood. However, reference datasets are not available for most other tissues, such as the kidney. In this study, we showed that the proportion of alterations attributable to changes in the cellular composition varies strikingly in the two disorders (asthma and systemic lupus erythematosus), suggesting that the contribution of cell subtype proportion changes to functional genomic properties can be disease-specific. We also showed that a reference dataset from a single-cell RNA-seq study successfully estimated the cell subtype proportions in mouse kidney and allowed us to distinguish altered cell subtype differences between two different knock-out mouse models, both of which had reported a reduced number of glomeruli compared to their wild-type counterparts. These findings demonstrate that testing for changes in cell subtype proportions between conditions can yield important insights in functional genomics studies.

## Introduction

Assays that test genomic function are used to understand the cellular and genetic differences in phenotypes between individuals. In human disease studies, we invariably test samples that are composed of mixed populations of cell subtypes when performing commonly-used functional genomic assays, including gene expression profiling and assays testing DNA methylation. To date, several cell type deconvolution approaches for genome-wide assays have been published, and applied to test for sample heterogeneity in gene expression [1–7] or DNA methylation [8–15], mostly often in studies of tumors or peripheral blood mononuclear cells (PBMCs) [2,6,7,9,12,13,15–17]. The influence of variability in cellular composition between samples on gene expression patterns has been recognized for decades [18], the associations between immune cell infiltration and prognosis of tumor have been well demonstrated [19–25], and innovative approaches to identify cell-intrinsic changes (those not attributable to cell subtype effects) have been developed [9,12,26–28]. Despite this, many studies still omit even passing consideration of the effects of cell subtype proportion when interpreting results of genome-wide assays. Furthermore, when the influence of cell subtype variation is included in the analysis of functional genomics studies, in most cases the cellular heterogeneity is treated as a nuisance variable, confounding the researchers’ ability to identify cell-intrinsic changes. By treating cell proportion variation as a nuisance variable to exclude, we fail to identify potentially interesting tissue compositional differences associated with disease phenotypes.

We have recently described our interest in understanding how to use functional genomic data in phenotypic association studies, not only testing for cellular reprogramming, reflected by cell-intrinsic functional genomic changes, as is typically studied, but also examining the generation of distinctive repertoires of cell subtypes in individuals with the distinctive phenotype, which we have described as polycreodism [29]. As each cellular model can be distinctively informative in understanding how a phenotype developed, they could both be considered valuable insights resulting from functional genomic studies.

We show in this report that re-analysis of several published studies using a cell subtype deconvolution approach yields many additional insights not described in the original publications. We initially focus on studies of peripheral blood leukocytes, and then show the potential for single cell RNA-seq to provide insights into cell subtype composition in less well-characterized tissues such as the kidney. We then compare these reference-based approaches with a more commonly-used analysis to account for variation in samples lacking reference panels of cell subtype properties, surrogate variable analysis (SVA) [30,31]. We test how SVA performs to account for cell subtype proportion effects and other sources of variability in the data studied. We conclude that cell subtype proportions themselves should be estimated as a specific goal of functional genomic studies, rather than discarded as a confounding influence, testing both the cellular reprogramming and polycreodism cellular models in phenotypic association studies.

## Results

### Datasets used in this study

We used publicly-available datasets from the Gene Expression Omnibus (GEO), focusing on studies of peripheral blood and on embryonic day 14.5 (e14.5) mouse kidney (**Table 1**). All datasets were assessed for quality, including the elimination of samples from further analysis when there was evidence of misidentification (for example, supposedly female samples expressing genes from the Y chromosome).

**Table 1:** Summary of datasets used in this study.

						Gene expression		DNA methylation	
Study number		GEO project identifiers	Study design	n	Authors	Analyzed tissue type	Assays	Analyzed tissue type	Assays
Study 1	Human	GSE69683	Severe asthma/healthy	422	Bigler et al. [32]	WB	Affymetrix HT HG-U133+PM	N.A.	
Study 2	Human	GSE82221	SLE/healthy	33	Zhu et al. [33]	PBMC	Illumina HumanHT-12 V4.0	WB	Illumina 450k infinium
Study 3	Human	GSE65219, GSE58888	Nonagenarian/ young	154	Nevalainen et al. [34]	PBMC	Illumina HumanHT-12 V4.0	PBMC	Illumina 450k infinium
Study 4	Mouse	GSE6287	Renal vesicle, s-shaped body, and collecting duct	8	GUDMAP database [35,36]	e14.5 kidney	Affymetrix Mouse Expression 430A	N.A.	
Study 5	Mouse	GSE4230	Lim1 conditional mutant mice	4	Chen et al. [37]	e14.5 kidney	Affymetrix Mouse Genome 430 2.0	N.A.	
Study 6	Mouse	GSE45844	Nephron progenitor-specific Sall1 deletion	6	Kanda et al. [38]	e14.5 kidney	Agilent Whole Mouse Genome	N.A.	

SLE, systemic lupus erythematosus; N.A., not analyzed; PBMC, peripheral blood mononuclear cell; WB, whole blood

### Cell subtype proportions influence gene expression results

Our first analysis was of gene expression profiles of human peripheral blood leukocytes, in a study initially designed to compare individuals with severe and moderate asthma and healthy controls [32]. The authors performed the expression analyses on the total RNA extracted from blood collected in PAXgene tubes using the PAXgene blood miRNA kit [32]. As variation in gene expression levels between individuals can be due to a combination of alterations of gene activity within cells (cell-intrinsic changes) as well as alterations of the proportions of cell subtypes in the sample, our goal was to understand the degree to which each mechanism was influencing the gene expression changes observed by the authors.

**Fig 1:**
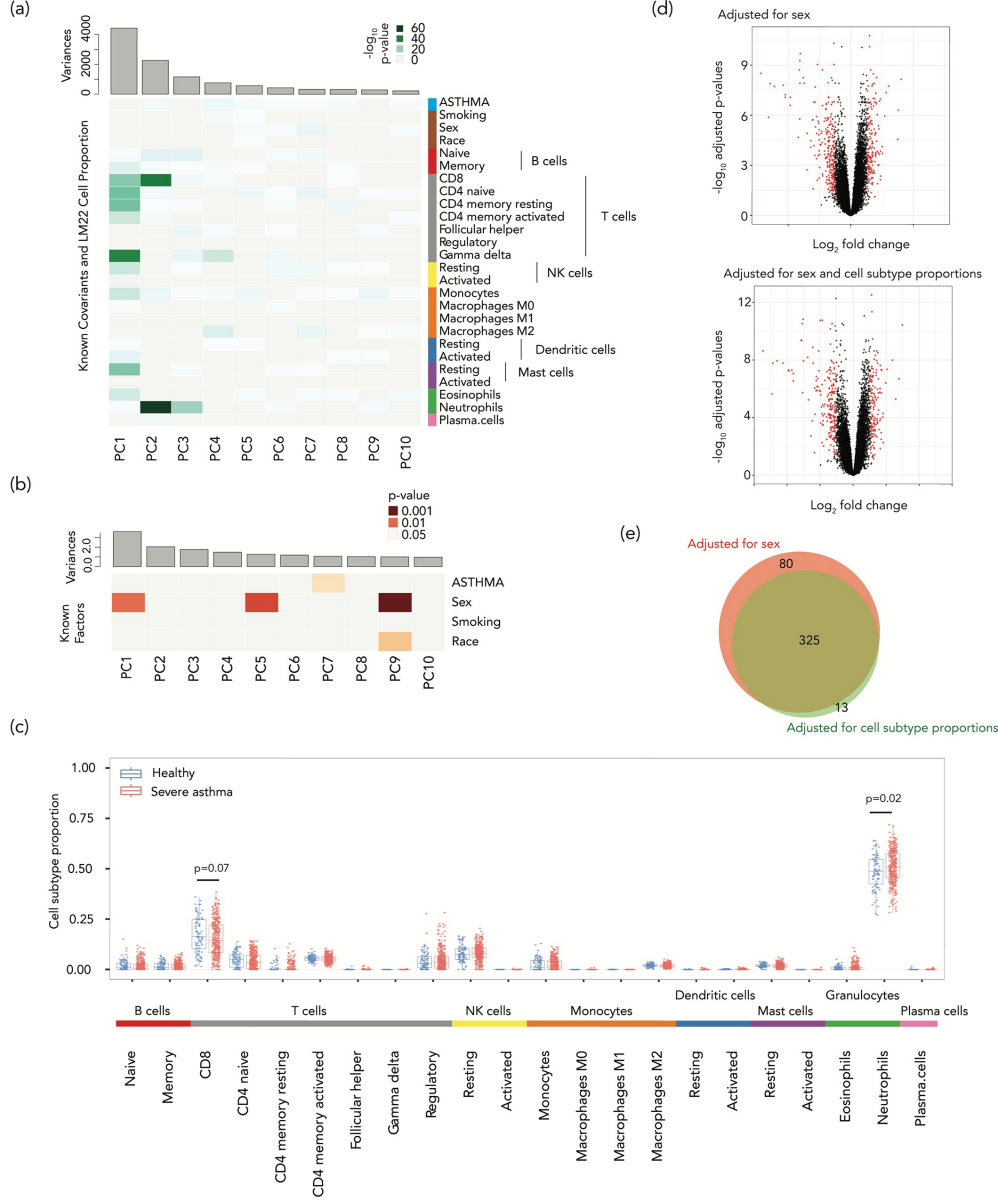
Deconvolution shows a strong effect of cell subtype proportions on gene expression variation in a study of blood leukocytes from asthmatics. (a) Principal components for gene expression with significance of association of different factors shown as a heat map. The disease status of severe asthma (ASTHMA) was very weakly associated with the variability in gene expression, accounting for only 0.31% of the first PC (which accounts for 20.9% of the variance, p = 0.25) and 4.1% of the second PC (10.68% of the variance, p = 2.9×10^−5^) of expression variation. (b) The same kind of analysis was performed but this time testing the contributions to the cell subtype proportional variability, with small contributions to principal component 1 (accounting for 16.54% of variance) of disease status (0.023%, p = 0.75) and sex (1.56%, p = 0.01). (c) Looking into why the cell subtypes were so influential in altering gene expression, we find the proportions of two cell types to be significantly different in patients with severe asthma, an increased proportion of neutrophils (p = 0.021, Mann-Whitney test) and a decreased proportion of CD8+ T cells (p = 0.072, Mann-Whitney test). In (d) we show two volcano plots, the upper showing 405 differentially-expressed genes (DEGs, FDR-adjusted p value <0.05, >1.2 fold change in expression) in red, representing those identified without cell subtype proportion adjustment. The lower volcano plot shows the 166 DEGs following adjustment for cell subtype proportions using the PCs most strongly reflecting the cell subtype effects on expression variation. (e) The Venn diagram shows the overlap between the genes identified as differentially-expressed when adjusted for sex (red) and after additional adjustment for cell subtype proportional variation (green).

We took advantage of the availability of reference expression profiles for 22 different subtypes of leukocytes (LM22) [5] and the *CIBERSORT* program, which uses a penalized multivariate regression approach to infer cell subtype proportions [6], allowing us to estimate blood cell subtype proportions in whole blood samples from 422 individuals, both patients with severe asthma (204 females, 130 males) and healthy controls (34 females, 53 males). We then performed a principal component analysis (PCA) to estimate the contributions to gene expression variability from disease status as well as sex, smoking, and race, in addition to the influence of each estimated cell subtype proportion (**Fig 1A**). To measure the contribution of each covariate, we used a linear modeling approach. The principal components (PCs) of variation of the expression profiles were modeled as a linear function of cell subtype proportions. Although disease status (severe asthma) was very weakly correlated with the first two PCs of gene expression variation (PC1 (20.9% of the variance), R^2^ = 0.0031 and p = 0.25, PC2 (10.68% of variance) R^2^ = 0.041 and p = 2.9×10^−5^), cell subtype proportion variation showed a much more significant influence on gene expression (**S1 Table**). These results indicate that for this asthma dataset, the primary determinant of gene expression variation was cell subtype proportion, potentially contributing to or reflective of the disease process.

### Cell subtype proportion changes in asthma

We then performed a further PCA to identify the factors in the phenotypic data and metadata that were most associated with the cell subtype proportions observed. The input, in this case, was the matrix of estimated cell subtype proportions across samples, and the objective was to find the most significant contributors to the variance in cell subtype proportions between samples. We tested these correlations and the significance of the contribution of each phenotypic variable to each PC of the expression profiles. We found no significant correlation with disease status (R^2^ = 0.00023, p = 0.75) and a small but significant contribution of sex (R^2^ = 0.01557, p = 0.011) to the first principal component of the estimated cell subtype proportions (16.54% of variance) (**Fig 1B**). We observed that the proportion of neutrophils was significantly increased in severe asthma patients compared to healthy controls (p = 0.02, Mann-Whitney test), with a decrease in the proportion of CD8+ T cells (p = 0.07, Mann-Whitney test) (**Fig 1C**). These results are consistent with several prior studies that have reported associations between neutrophils and asthma severity [39–43]. Less is known about the role of CD8+ cells in asthma. While some studies have reported fewer CD8+ T cells in allergic asthma [44], others have found the interferon-producing CD8+ T cells were associated with greater asthma severity and not atopy [45]. Since atopy is associated with fewer CD8+ T cells [46], and most participants in this study were atopic (of the 334 severe asthmatics, atopy information was available for 308, of whom 234 were positive (76.0%); of the 87 controls, atopy information was available for 76, of whom 32 were positive (42.1%)), their atopic status rather than their asthma may explain the reduced CD8+ proportions. Our re-analysis of functional genomics data associates severe asthma in atopic individuals with significantly higher neutrophil and lower CD8+ T cell proportions, which offers potential insights into the cellular events occurring in these individuals. These results suggest that the variation of cell subtype proportions may not be merely a confounding variable in gene expression studies but can potentially contribute useful insights into the biological processes occurring in a disease.

### Cell-intrinsic gene expression changes in asthma

When the effects of cell subtype proportional changes are eliminated, the changes in gene expression that remain are more likely to represent altered levels of gene transcription within the cells tested. We borrow a term used in the study of DNA methylation to refer to these as ‘cell-intrinsic’ gene expression changes [47], reflecting what we have also described as ‘cellular reprogramming' [29]. Including and adjusting for sex as a covariate in our analyses, we identified 405 differentially-expressed genes (DEGs) without adjusting for cell subtype proportions (false discovery rate (FDR)-adjusted p-value <0.05, >1.2-fold change in expression, **Fig 1D**, genes listed in **S2 Table**). When we performed an adjustment for cell subtype proportions including each of the individual cell proportion values in our linear model, as is typically performed in studies of DNA methylation [48–50], only 142 genes remained categorized as DEGs (listed in **S3 Table**). However, this approach is not ideal, as it introduces a large number of covariates into a multi-variable linear regression model, and these covariates are collinear with each other (as one cell subtype proportion goes up, other proportions have to go down). We therefore used the alternative approach of regression on PCs of cell subtype proportions [51,52] using the PCs that most strongly reflected the cell subtype proportion effects on expression variation (those with a p<0.01 and explaining >1% of the variation of cell subtype proportions: PCs 1–5 and 9 in **S1 Fig**). We thus reduced the dimensions of the covariates and eliminated their collinearity. This PC-based approach now defined 338 genes to be differentially-expressed (**Fig 1D**, genes listed in **S4 Table**), eliminating 80 of the 405 DEGs initially identified prior to cell subtype adjustment, and adding a further 13 genes not previously recognized to be differentially expressed (**Fig 1E**). As would be expected, more than 85% of the DEGs eliminated by this approach have evidence for being expressed in specific blood cell subtypes [53–56] (**S5 and S6 Tables**). The 13 newly identified DEGs showed higher variances compared to eliminated DEGs (p<0.001, permutation test).

### Cell subtype effects are dominant in SLE

**Fig 2:**
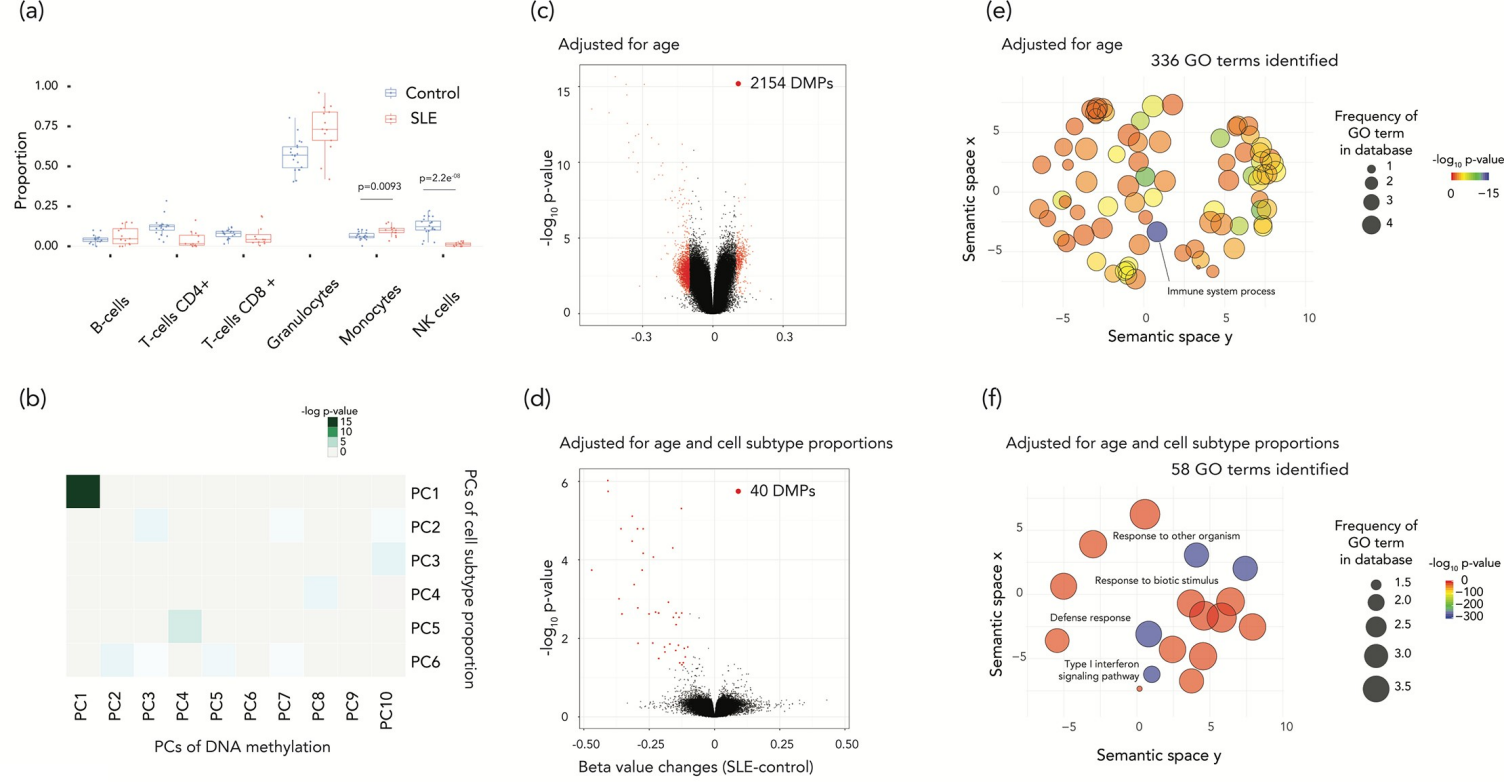
Disease status associated cell subtype proportion changes reflect the DNA methylation changes between SLE and control samples and the disease-related pathways were highlighted after the cell subtype proportion adjustment. (a) The cell subtype proportion changes in SLE were driven predominantly by two cell types. The proportion of monocytes in increased in SLE (p = 0.0093, t-test). On the other hand, the proportion of natural killer (NK) cells is lower in patients with SLE (p = 2.2e^-08^, t-test). (b) Principal components for DNA methylation with significance of association of PCs for the estimated cell subtype proportions are shown as a heat map. The first principal components were significantly associated each other. (c-d) Two volcano plots show differentially-methylated probes (DMPs). (c) shows the results of DMPs adjusted for age alone and (d) shows results also adjusted for cell subtype proportions. Almost all DMPs were eliminated after the adjustment for cell subtype proportions. (e-f) GO analysis results are summarized as REVIGO scatterplots. (e) shows the results of age-adjusted and (f) the results for age and cell subtype proportion adjusted terms. The x and y axes indicate the semantic similarity of each GO term. The bubble color indicates log_10_ p-values, and size represents the percentage of genes annotated with the GO term in the human database.

Systemic lupus erythematosus (SLE) is an autoimmune disease caused by cells represented in the peripheral blood circulation [57]. We used a cross-sectional study which tests the DNA methylation alterations in whole blood and transcription alterations in circulating PBMCs of 30 SLE patients, including 15 with lupus nephritis (LN) (SLE LN+), 15 without LN (SLE LN-), and 25 normal controls (NC) [33]. We used NC and SLE LN- samples in our study. Our cell subtype deconvolution revealed that the proportion of monocytes was significantly increased and the proportion of resting natural killer (NK) cells was significantly decreased in SLE, obtaining the same results using either DNA methylation or gene expression data (**Fig 2A and S2 Fig**). These cell subtype proportion changes revealed by functional genomic data are consistent with prior literature describing a lower proportion of NK cells and a higher proportion of monocytes in patients with SLE [58]. In addition, 53.8% of the first PC of DNA methylation variation (which accounted for 16.3% of the variance, the linear model p-value = 9.1e^-14^) and 94.9% of the second PC (10.6% of variance, the linear model p-value = 0.084) were attributable to cell subtype variation (**Fig 2B)**. Similar results were obtained from gene expression estimates of cell subtype proportions, with 78.9% of the first (accounting for 12.5% of the variance, p = 0.012) and 81.8% of the second principal component (9.1% of the variance, p = 0.005) attributable to cell subtype variation (**S2 Fig)**. When we re-analyzed gene expression differences between SLE and control subjects having accounted for cell subtype variability (using gene expression information), we found that only 4 genes remained significantly differentially-expressed out of the 485 DEGs (false discovery rate (FDR)<0.05 and log_2_ fold-change (FC)>1.2, **S7 Table**) originally identified without adjusting for cell subtype proportions. In the DNA methylation analysis, we identified 2,154 differentially methylated CGs (FDR<0.05, 𝛿beta(case-control) ≥10%, at 1,366 genes) before adjustment, but just 40 CGs (at 27 genes) after adjusting for cell subtype proportions (**Fig 2C and 2D and S8 Table)**. This suggests that almost all significant differences in gene expression and DNA methylation may be attributed to systematic cell subtype proportion variation between individuals with SLE and healthy controls. When we linked the 40 CGs with the 27 nearby genes (using the Illumina microarray design annotation) and tested gene ontology (GO) terms for enrichment, the list of most significantly enriched GO terms changed compared with the unadjusted data (**Fig 2E**). The new terms included a strong enrichment for type I interferon signaling pathways (**Fig 2F**), revealing a known pathogenic mechanism in SLE now being targeted therapeutically [59].

### Evaluation of a reference-free approach which adjusts for nuisance variables on cell subtype effects

**Fig 3:**
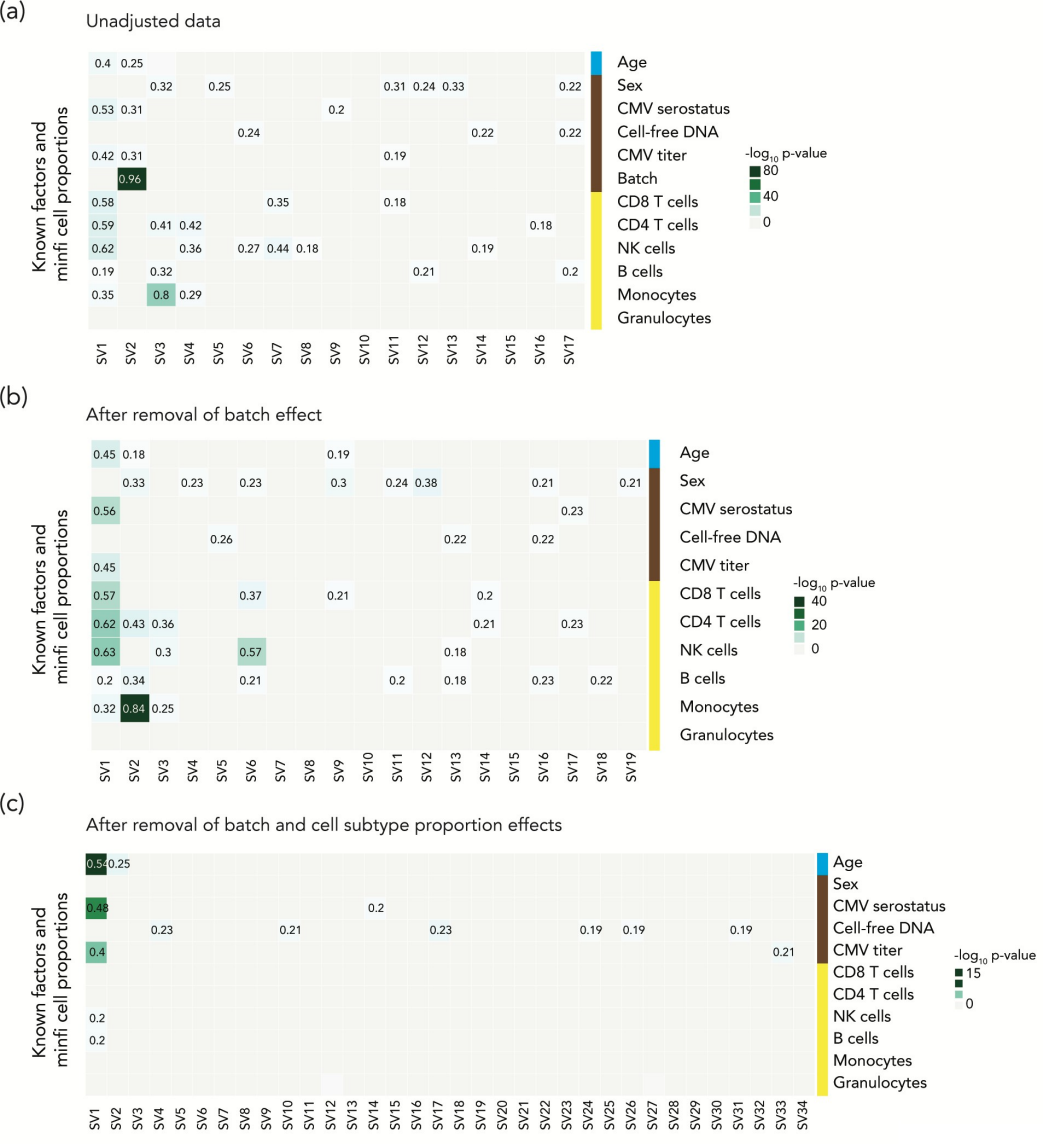
The SVA approach may lead to elimination of true positive results. Heatmaps show the influences on each surrogate variable (SV) of known covariates and estimated cell subtype proportions. We performed SVA on the unadjusted data (a), and on data after removal of batch effect (b) and after further adjustment for cell subtype proportion variability (c). We performed multiple linear regression models to estimate the contribution of covariates to surrogate variables. The step-wise analysis revealed that the SVA approach has effects on the phenotype of interest, especially as we eliminate some of the sources of experimental artefact.

The alternative to reference-based deconvolution approach is to account for heterogeneity influencing the signal, using an approach like Surrogate Variable Analysis (SVA), which is probably the most commonly used approach in DNA methylation studies [60]. SVA is an attractive choice, as it should allow the data to be adjusted for any type of confounder including the effects of cell subtype proportion heterogeneity as well as influences like batch effects. Therefore, it has been recommended and used for the sample types for which there is incomplete knowledge of cell-type composition or the presence of unknown confounders [61,62]. We explored how SVA performs in the unusual situation when insights are available from deconvolution into cell subtype composition. We studied the DNA methylation data from a study of aging (GSE58888; 122 nonagenarians and 21 young controls (19–30 years)), testing how each surrogate variable was influenced by each of the metadata variables such as sex, cytomegalovirus infection (serostatus and titer), cell-free DNA level or batches. In **Fig 3A** we show that SVA does indeed predict cell subtype proportions as surrogate variables, proportions that we estimated using Houseman’s reference-based deconvolution approach [12,63,64], as well as picking up a strong influence of experimental batch (R = 0.96). However, despite defining age as the phenotype of interest, we find that the SVA also recognizes this as a source of variability (R = 0.4), which would result in an unrecognized loss of the signal sought in these studies. To simulate a situation in which cell subtype proportion and batch effects are not major confounding influences in an experiment, we re-processed the aging data to remove batch effects on their own or in combination with cell subtype proportion effects. In those situations, the effect of SVA to capture the phenotype of interest, age, gets progressively stronger (**Fig 3B and 3C**). SVA therefore disproportionately affects what would be better-executed studies with fewer cell subtype and technical artefacts.

### Single-cell RNA-seq data for cell subtype deconvolution

**Fig 4:**
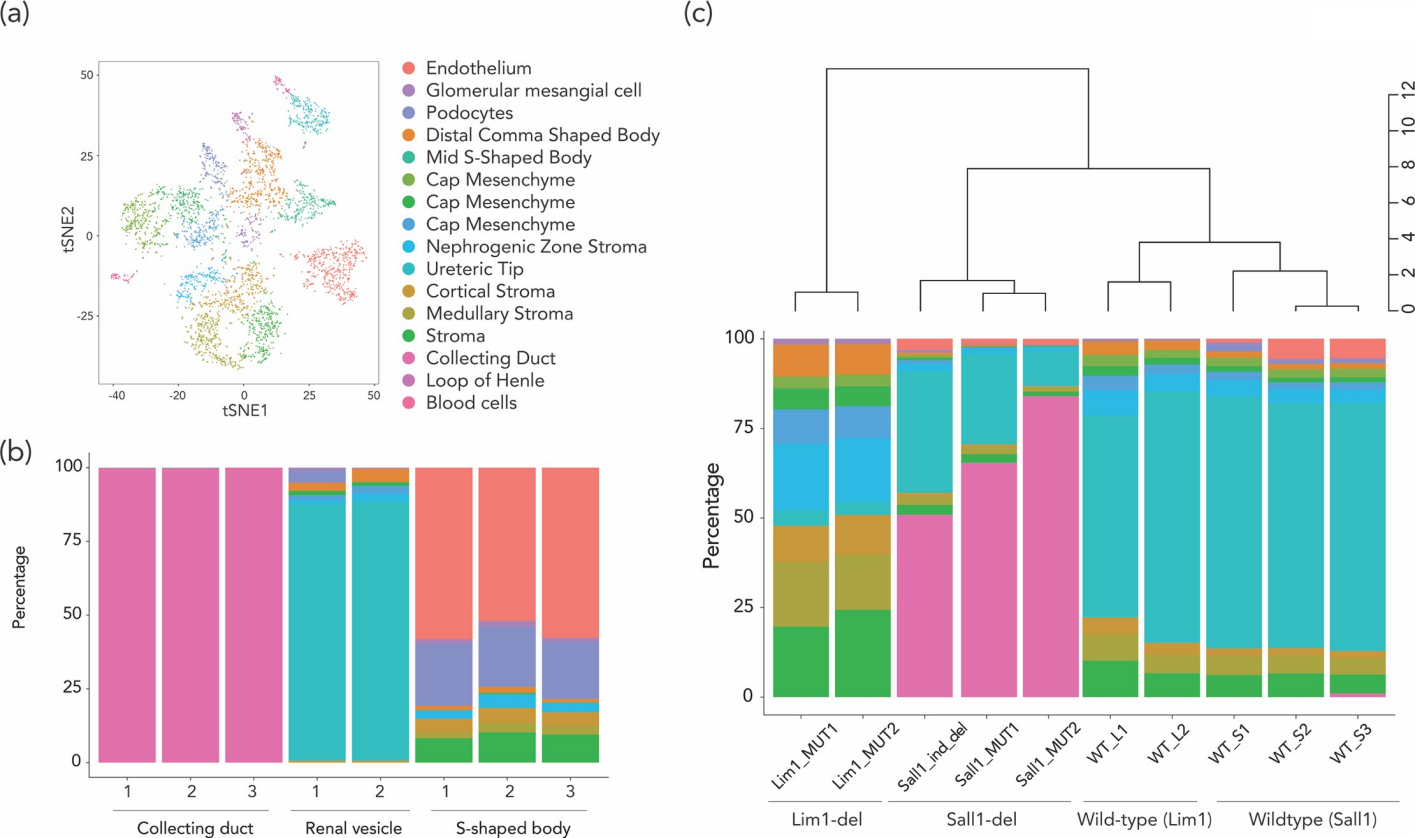
The estimation of cell subtype proportion using e14.5 mouse kidney scRNA-seq data showed the applicability of scRNA-seq data for the deconvolution approach. (a) The scRNA-seq analysis of e14.5 mouse kidney identified 16 cell type clusters in total. We performed cell subtype proportion estimate on (b) microdissected samples (collecting duct, renal vesicle, and s-shaped body) from e14.5 kidney and (c) conditional knockout mouse models.

Tissues like the hematopoietic system composed of individual cells with well-characterized surface markers are relatively easier to characterize in terms of cell subtypes compared with the cell subtypes in solid tissues. To perform cell subtype deconvolution using reference gene expression or DNA methylation data with solid tissues would require large amounts of tissue, and generation of informative surface markers to sort the cell subtypes. As an alternative, single-cell RNA-seq (scRNA-seq) can test the gene expression patterns in different cell subtypes in a tissue without the need to isolate the cell subtypes. The cell subtype-specific genes defined by scRNA-seq data [65] can be used to create reference panels of genes that can then be used for deconvolution of bulk RNA-seq data. To test this approach, we used an e14.5 mouse kidney scRNA-seq data set [66], analyzed using Seurat [65], identifying 16 cell subtype clusters in total (**Fig 4A**). Based on the expression status of known cell subtype-specific genes, we identified the cell subtypes corresponding to each cluster by known cell subtype-specific genes [66] (**S9 Table**). The cell subtype-specific genes were identified using the *FindAllMarkers* function of Seurat (requiring ≥30% of cells in the cluster to be expressing the genes, with the fold change threshold = log_2_(1.5)), following which the median expression of the gene in each cell subtype was calculated. After eliminating ribosomal, mitochondrial and sex chromosome genes, we identified 722 cell subtype-specific genes in total. The resulting cell subtype-specific gene expression signature was used as a reference for estimating cell type proportions by CIBERSORT (cell subtype-specific genes in **S10 Table**). We first applied this information to gene expression data from three microdissected kidney tissue components (renal vesicle, S-shaped body, and collecting duct, GUDMAP Series ID 8, GSE6287). Using the deconvolution of gene expression, >99.9% of the cells in the collecting duct samples were identified as collecting duct cells. In the microdissected renal vesicle samples, the dominant cell type was ureteric tip cells, while the microdissected S-shaped body consisted of endothelium, podocytes, cap mesenchyme and stromal cells, all findings that would be expected from these kidney sub-structures (**Fig 4B**). We then used the deconvolution approach on bulk gene expression data of e14.5 mouse kidneys from two conditional knockout mouse models (nephron progenitor-specific Sall1 deletion [38] (Sall1-del), and Lim1 metanephric mesenchyme-specific conditional mutant [37] (Lim1-del)). Both mouse models had been noted to have decreased numbers of nephrons, but our cell subtype studies revealed otherwise distinctive patterns. The Sall1-del mice had a dramatically reduced proportion of renal cortical cells, with a corresponding increase in collecting duct cells (**Fig 4C**). Histologically, these mice showed multiple glomerular cysts, dilated tubules, thin cortex, and significant developmental impairment of nephron contents, consistent with the inferred lack of renal cortical cells. The Lim1-del mice had been shown to have almost no nephrons histologically [67]. This is consistent with the results of our deconvolution analyses, which showed almost complete loss of ureteric tip cells (the dominant cell subtype of wild-type kidneys, and of microdissected renal vesicle samples), with replacement by stromal cells (nephrogenic, medullary, and cortical) as the primary remaining cell subtype of the Lim1-del mouse kidney (**Fig 4C**). These mouse results support the possibility that scRNA-seq profiles can be used accurately for cell subtype deconvolution in solid tissues.

## Discussion

By using assays that test expression of genes or microRNAs, the methylation of DNA, chromatin states or other indicators of genomic function, we are generally trying to understand the innate characteristics of the cells tested. Such cell-intrinsic changes can reflect responses to environmental perturbations or genetic mutations, and can be used as clues to the pathogenesis of an associated phenotype. We have referred to this as cellular reprogramming [29], the alteration of the molecular characteristics of a canonical cell type. The possibility that cell subtype proportional heterogeneity could be contributing to the variability in the results of the functional genomics assay is not always considered, but when addressed is generally treated as a confounding variable, with the focus on cell-intrinsic changes of functional genomic properties.

We have pointed out that the systematic alteration of cell fate decisions and the repertoire of cell subtypes in tissue is a potential outcome of transcriptional regulatory perturbations, potentially contributing to the development of specific phenotypes, an alternative cellular epigenetic model that we have called polycreodism [29]. In the current study, we sought to understand the relative contribution of cellular reprogramming and cell subtype proportion changes on gene expression and DNA methylation changes associated with different types of phenotypes, from physiological studies of aging and the disease phenotypes of asthma and systemic lupus erythematosus, as well as mouse conditional knockout models. Our focus was on studies of two tissue types: 1) peripheral blood leukocytes, not only because of the technical advantages they offered for our cell subtype deconvolution approaches, but also because many transcriptomic studies and most published large-scale epigenome-wide association studies (EWAS) testing DNA methylation have been performed on blood cells, and 2) kidney, which is a highly complex organ with relatively less characterization of individual cell subtypes. By gaining insights into the relative contributions of cellular reprogramming and polycreodism in each condition, we contributed to the interpretability of these prior studies that did not take in account for cell subtype proportion variation.

Our studies were based on the ability to estimate cell subtype proportions from gene expression or DNA methylation data. It was helpful to have reference gene expression and DNA methylation data on purified peripheral blood leukocytes [6,12]. Using these datasets, we could readily estimate the cell subtype proportions of the samples tested. From the asthma study which used whole blood samples, we found that 80 DEGs out of 405 DEGs were eliminated after the cell subtype proportion adjustment, and more than 80% of those genes were expressed in a cell-type specific way. Therefore, those variations in gene expression are attributed to cell subtype proportion variations between severe asthma and healthy controls. On the other hand, while the 13 newly identified DEGs are also correlated with cell subtype proportions, they showed higher variability in expression between samples than the genes eliminated for being related to cell subtype proportion variation alone. This finding suggests that some cell subtypes have differential expression of genes between asthma and controls. We note that in tissues other than blood such reference gene expression and DNA methylation data are unlikely to be readily available, prompting us to explore whether scRNA-seq data could be used in these deconvolution studies. Our reference gene expression signatures derived from scRNA-seq data from the e14.5 mouse kidney was applied to microdissected samples and conditional mouse knockout data. Our results demonstrated that the estimated cell subtype proportions based a reference gene expression signatures derived from scRNA-seq data showed concordance with microdissected samples. In conditional mouse knockout studies, we successfully predicted each case in which cell subtypes were expected to be over- or under-represented in each sample from the original analysis. For example, although the authors could detect that deletion of Lim1 in metanephric mesenchyme-derived tissue downregulated nephron-specific genes using a conventional differential gene expression analysis [37], our re-analysis revealed that the deletion leads to severe loss of ureteric tip cells, but mesenchyme cells surrounding ureteric tips remain present, and the lost proportion was replaced by stromal cells. Their histopathological analysis using LacZ transgenic animals [67] suggested that metanephros growth and ureteric bud branching were relatively normal but nephrons were completely absent in the mutant. The authors speculated that the absence of Lim1 function could result in the loss of a subset in the renal vesicle. Combining their results and our re-analysis, we show that the loss of Lim1 in metanephric mesenchyme-derived tissue prohibits ureteric tip formation in the renal vesicle. The Six2-dependent Sall1 depletion mice also showed a reduction of nephron numbers; however, this alteration is due to reduction of cap mesenchyme and nephrogenic zone stroma, not due to deletion of the ureteric tip as found in the Lim1 mutation, resulting in the collecting duct becoming the major remaining cell type. These findings indicate that two distinct mouse models, both involving nephron depletion, showed model-dependent cell subtype loss incompletely characterized by orthodox analyses looking for differential expression of genes. Our results strongly suggest that scRNA-seq data, which can be generated from scRNA-seq or will be imminently available from public datasets like the Human Cell Atlas (https://www.humancellatlas.org), especially for less well-characterized solid tissues, will be a helpful way of understanding the cell subtype proportion source of variation in functional genomic assays of such tissues.

There were other observations made that are of technical importance for performing functional genomics studies. We were concerned that using the SVA approach had the potential to mask some of the genuinely phenotype-associated effects, especially in better executed studies. We note the strong concordance of results when adjusting for cell subtype proportions using gene expression and DNA methylation data. This indicates that deconvolution using results of one functional genomics assay can be used to adjust for cell subtype proportions when analyzing a completely different kind of assay of the same samples. This would highlight the applicability of usage of a reference panel based on data from scRNA-seq analysis, which is becoming a popular method. We were also careful to avoid using the individual cell subtype proportions in the multivariable linear regression model, as they can be numerous and are inherently collinear, instead of using regression on PCs [51,52], choosing the PCs capturing most of the effects of cell subtype variability. These insights should be generally useful when performing and analyzing epigenetic association studies in particular and functional genomic assays in general.

Instead of focusing on the cell-intrinsic alterations, as would be typical, we generated two outputs from the functional genomics studies. The first was a high-confidence set of genes or loci undergoing alterations in gene expression or DNA methylation, manifesting changes that could not be attributed to cell subtype proportional variability, indicating cellular reprogramming effects. The second was the difference in cell subtype proportions between the comparison groups. This is not typically an output of analytical approaches used for gene expression or DNA methylation studies, but was explicitly sought in our analytical approach, and revealed systematic changes. It should be relatively straightforward to modify excellent software packages such as *minfi* [13] to allow this additional output to be generated routinely. In particular, the study of SLE was striking for having an overwhelming effect of cell subtypes on gene expression and DNA methylation variation. While this might currently be considered a negative result, if cell subtype proportions are treated purely as confounding variables, we note that the SLE patients had distinctive NK cell and monocyte proportions, which represents the use of functional genomic data to gain an insight into cellular events contributing to the disease process. These cellular changes have already been recognized independently in SLE, with decreased NK cell activity correlating with active disease and observed to a greater extent among those with renal involvement [68–71]. Conversely, more activated monocytes have been found in individuals with SLE [72,73] associated with disease complications such as atherosclerosis [74]. We note that the cell composition changes were not limited to SLE. We observed cell subtype proportion changes in all studies we examined, including in asthma and aging. Aging is strongly associated with T cell proportion changes [75–79], and immune cell proportion changes including neutrophils [39–43], T cells [80–82] as well as eosinophils [83–85] are also well-reported in the asthma patients. Therefore, these results underscore the value of looking simultaneously for cellular reprogramming and cell repertoire changes in functional genomics studies, as each can be harvested from the functional genomics data generated and can be valuable in providing insights into the condition being studied. Surrogate variable analysis which does not require a reference panel, on the other hand, eliminate this useful information, another reason for caution in choosing approaches such as SVA.

We conclude that, while it should not be surprising that cell subtype proportions need to be taken into account in the interpretation of functional genomics studies of heterogeneous samples, variability of cell subtype composition can also provide insights into the phenotype being tested, and should not be discounted as merely a confounding factor [12,86–88]. Phenotypes may indeed result from cellular reprogramming, but it is highly plausible that the polycreodism model of altered cell repertoires in tissue is another potentially very powerful mechanism for mediation of phenotypic changes. By testing simultaneously for the cellular models of reprogramming and polycreodism, we increase our capacity for the discovery of new insights into the pathogenesis of diseases or the development of other phenotypes.

## Methods

### Dataset used in this study and preprocessing data

All datasets used in this study are published and publicly available through the Gene Expression Omnibus (GEO, https://www.ncbi.nlm.nih.gov/geo/), from which we downloaded the datasets. The GEO accession numbers and study designs are described in **Table 1**. Phenotypic data were extracted from the matrix tables provided by the authors on the GEO website. Before our re-analysis, we tested the quality of data, including batch effects and possible sample swapping, excluding samples when the information about sex provided by the authors was discordant with the data obtained from the sex chromosomes. For the DNA methylation datasets, we first filtered out poor quality samples by testing detection p-value distributions to see background noise level and eliminating samples with high background (average detection p-values >0.01), and by performing PCA, which found a single sample to cluster very distinctly from all of the others, causing us to remove it from further analysis. We then performed quantile normalization using the *preprocessQuantile()* function in the *minfi* R package [13], and filtering out probes (a) have failed to hybridize (detection p-values >0.01), (b) probes overlapping with and around known single-nucleotide polymorphisms (SNPs) and 1000G SNPs (minor allele frequency (MAF) >0.1), (c) probes that have been shown to be cross-reactive[89], and (d) probes on sex chromosomes (except study 4, for which we only used female samples). For expression datasets, we aggregated each transcript value by HUGO gene symbol to calculate mean expression values for each gene. We describe these results in detail in the **S1 File**.

### Reference-based estimation of cell subtype proportions

We estimated cell subtype proportions based on gene expression and DNA methylation data. From the DNA methylation profiles, we estimated the proportions of CD8+ T cells, CD4+ T cells, NK cells, B cells, monocytes and granulocytes using *the estimateCellCounts()* function from the *minfi* R package [13], which is modified from the original Houseman reference-based approach [12]. From gene expression profiles, we ran *CIBERSORT* [6] using two different signature gene files, the *CIBERSORT* default file based on expression profiles from 22 leukocyte subtypes (LM22) [5], and a signature gene profile generated from publicly-available scRNA-seq results from 68,000 PBMCs [90] using the *Seurat* R package[65].

### Associations between cell subtype proportions and phenotype

We performed principal component analysis (PCA) on the cell subtype proportion estimates obtained. We tested for possible confounding influences using metadata provided by the study authors as a matrix table, including technical (batch, sample collection date) and biological (age, sex, phenotype) influences, using a linear modeling approach. We identified significant confounding covariates using ANOVA.

### The contribution of cell subtype proportion to functional genomic data

We performed PCA on gene expression values (aggregated expression values) and DNA methylation values (quantile normalized M values), then we tested the contribution of cell subtype proportions to each principal component (PC) using a linear modeling approach. The degree of contribution to each PC was estimated by the R-squared of the regression model, and the significance of each was tested using ANOVA.

### Identifying gene expression and DNA methylation changes

To identify differentially methylated probes (DMPs) and differentially expressed genes (DEGs), we performed regression analysis with the lmFit function of the limma R package using the M values of DNA methylation data and the log-transformed values of expression data [91]. We selected biological covariates provided by the authors to be included into the model based on data from each PCA. We built models with and without cell subtype proportion adjustments to test the effects of variability of cell subtype proportions. To avoid collinearity and high dimensionality of cell subtype estimates [92], we used a principal component regression instead of a linear regression approach using the actual cell proportions. The PCs we included in the linear model are those with significant associations with DNA methylation or expression variation (p-value <0.01) and which explain >1% of the variation of the cell subtype estimate. To identify significant DMPs, we retained the CpGs with FDR<0.05 and absolute beta value changes >10%. The DEGs were defined as the genes with FDR<0.05 and absolute fold changes of expression for studies asthma and SLE of >log_2_(1.2) and >log_2_(1.5) for the aging study using the same fold-difference threshold as the original publications. The proportional Venn diagrams were plotted using BioVenn [93].

### Gene ontology analyses

To identify the enriched gene ontology (GO) terms in the DMPs, we performed GO analysis using the Bioconductor package *GOseq* [94]. We used DMP corresponding gene symbols for searching enriched GO terms in the human hg19 database. We selected the terms which false discovery rate (FDR) adjusted p-values were less than 5% as significant GO terms. We performed the analysis on both with and without adjusting for cell subtype proportions. The significant GO terms were visualized using REVIGO [95], using the program’s default settings (*Homo sapiens* database).

### Surrogate variable analysis

We performed surrogate variable analysis (SVA) using the R package *sva* [30,31]. We selected the phenotype of interest information (young control or nonagenarians) for the analysis. We obtained 17 surrogate variables (SVs) on raw data, 19 SVs after the batch effect adjustment, and 34 SVs after adjustment for batch and cell subtype proportion effects. We describe these results in detail in the **S2 File**. We tested the correlations to known and estimated cell subtype proportions using mixed linear regression analysis. We included the SVs in the linear model to test the effects on DNA methylation status. To identify significant DMPs, we retained the CpGs with FDR<0.05 and absolute beta value changes >10%.

### Single-cell RNA-seq analyses

We downloaded the e14.5 mouse kidney scRNA-seq datasets (Drop-seq and Chromium 10x Genomics (10x Genomics)) [66] and analyzed the scRNA-seq data using the *Seurat* R package [65]. The Drop-seq data contains 22,939 genes in 200 cells, and the 10x Genomics data contains 27,998 genes in 2,295 cells. After filtering out the genes with fewer than three cells expressing the gene and cells in which fewer than 1000 genes were found to be expressed, we merged two datasets using canonical correlation analysis. The merged data contains 19,592 genes in 4,175 cells with a median number of detected genes per cell of 2,628 (standard deviation = 920.3). We then performed PCA for linear dimensional reduction. We identified 16 clusters in total, corresponding to 722 signature genes with distinctive expression status compared to other clusters, with on average at least 1.5-fold differences between the clusters compared with other clusters, and with at least 30% of the cells in the cluster expressing the gene using the *FindAllMarkers* function of the *Seurat* R package [65]. We calculated the median expression values of the signature genes in each cluster to generate a cell subtype signature profile for *CIBERSORT* analysis. We describe these results in detail in the **S3 File**. We provide the lists of known reference gene expression and signature genes expression of each cluster in **S9 and S10 Tables**.

## Supporting information

S1 Fig A correlation heatmap of the principal components (PCs). The PC of variation in cell subtype proportions (y axis) are correlated with the PCs for gene expression (x axis) in a study of asthma. In particular, the first and second PCs of cell subtype proportions are significantly associated with the PCs of gene expression. The PCs selected for adjustment are shown in red.
(TIF)

S2 Fig Deconvolution of cell subtype proportions from gene expression profiles of SLE patients and healthy control. (a) The estimated cell subtype proportions using gene expression status. A boxplot showed that proportions of T-cells regulatory and monocytes were significantly increased and NK-cells resting was decreased in SLE patient. The significance was calculated with Student t-test. (b) Principal component analysis for gene expression showed significant association with PCs of cell subtype proportions estimated from expression data. The significance was calculated by a regression approach. These results suggest that the gene expression variations also strongly correlated to cell subtype proportion variations. We selected PCs with significant associations with expression variation (p-value <0.01) and which explain >1% of the variation of the cell subtype estimate for cell subtype proportion adjustment. The PCs selected using these criteria are shown in red.
(TIF)

S1 Table Contributions of each cell type for top 5 principal components obtained by PCA on variation of the expression profiles (PC-ex). (XLSX)

S2 Table A list of differentially expressed genes without cell subtype proportion adjustment (Healthy-Severe Asthma). (XLSX)

S3 Table A list of differentially expressed genes with cell subtype proportion (actual cell proportion) adjustment (Healthy-Severe Asthma). (XLSX)

S4 Table A list of differentially expressed genes with PCs (PC1-PC5 and PC9) of cell subtype proportion adjustment (Healthy-Severe Asthma). (XLSX)

S5 Table A list of cell type-specific gene from PBMC scRNA-seq data. (XLSX)

S6 Table Lists of cell subtype proportion adjustment eliminated genes and newly identified genes. (XLSX)

S7 Table Lists of differentially genes in LUPUS patients before and after the cell subtype proportion adjustment (PC-ces). (XLSX)

S8 Table Lists of differentially methylated CpG site in LUPUS patients before and after the cell subtype proportion adjustment (PC-ces). (XLSX)

S9 Table A list of known reference gene expression profile of each cluster and the cell type estimate. (XLSX)

S10 Table A list of cell type specific genes in e14.5 mouse kidney. (XLSX)

S1 File Quality checks, preprocessing and estimating cells subtype proportions. (PDF)

S2 File Surrogate variable analysis. (PDF)

S3 File Generating an e14.5 mouse kidney signature profile from single cell RNA-seq results. (PDF)
