# Supplementary figures and images for: Insights from deconvolution of cell subtype proportions enhance the interpretation of functional genomic data
Source: PLoS One. 2019 Apr 25;14(4):e0215987. doi: 10.1371/journal.pone.0215987 (PMC6483354; doi:10.1371/journal.pone.0215987)

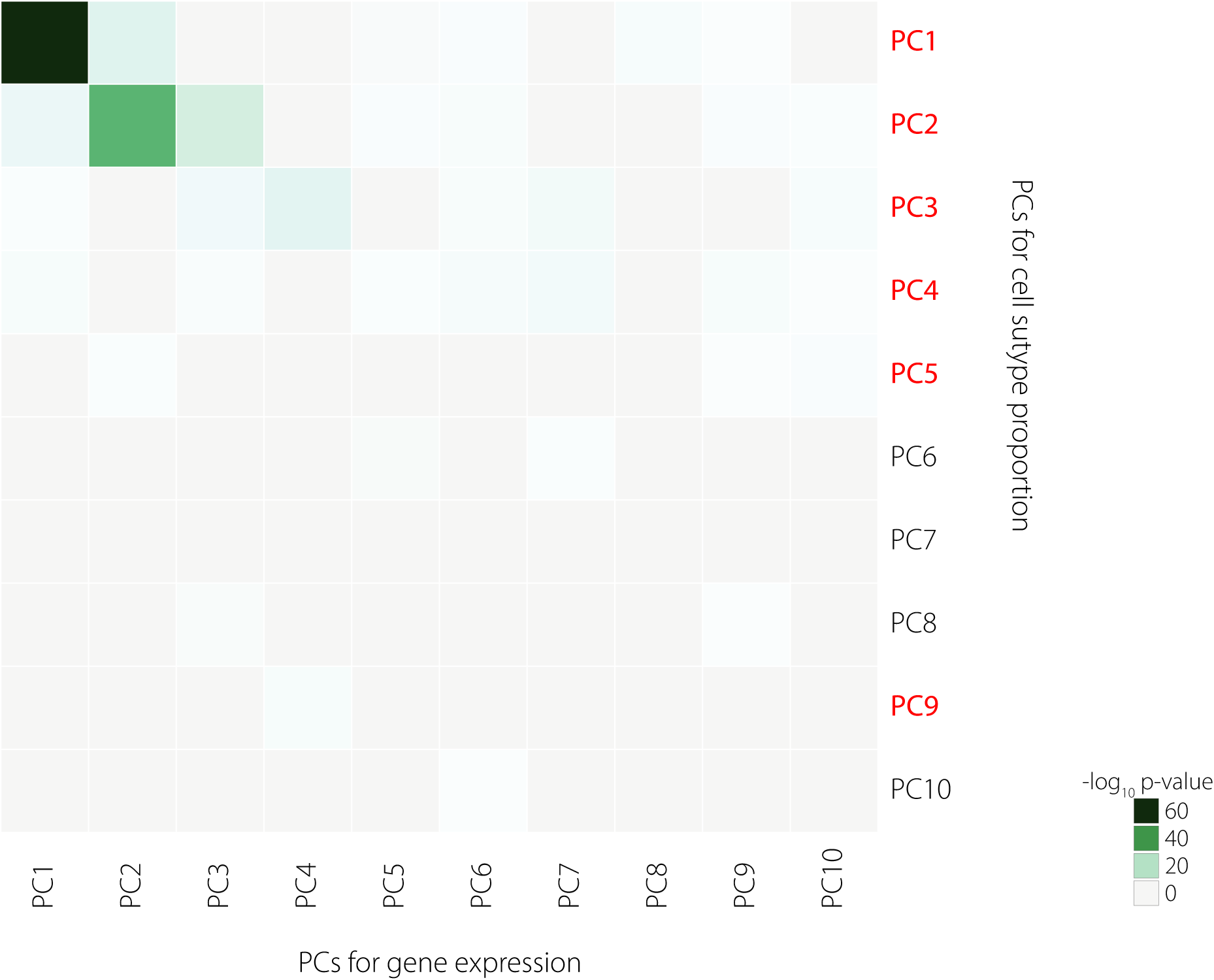

Supplement: S1 Fig — The PC of variation in cell subtype proportions (y axis) are correlated with the PCs for gene expression (x axis) in a study of asthma. In particular, the first and second PCs of cell subtype proportions are significantly associated with the PCs of gene expression. The PCs selected for adjustment are shown in red. (TIF) [file pone.0215987.s001.tif]

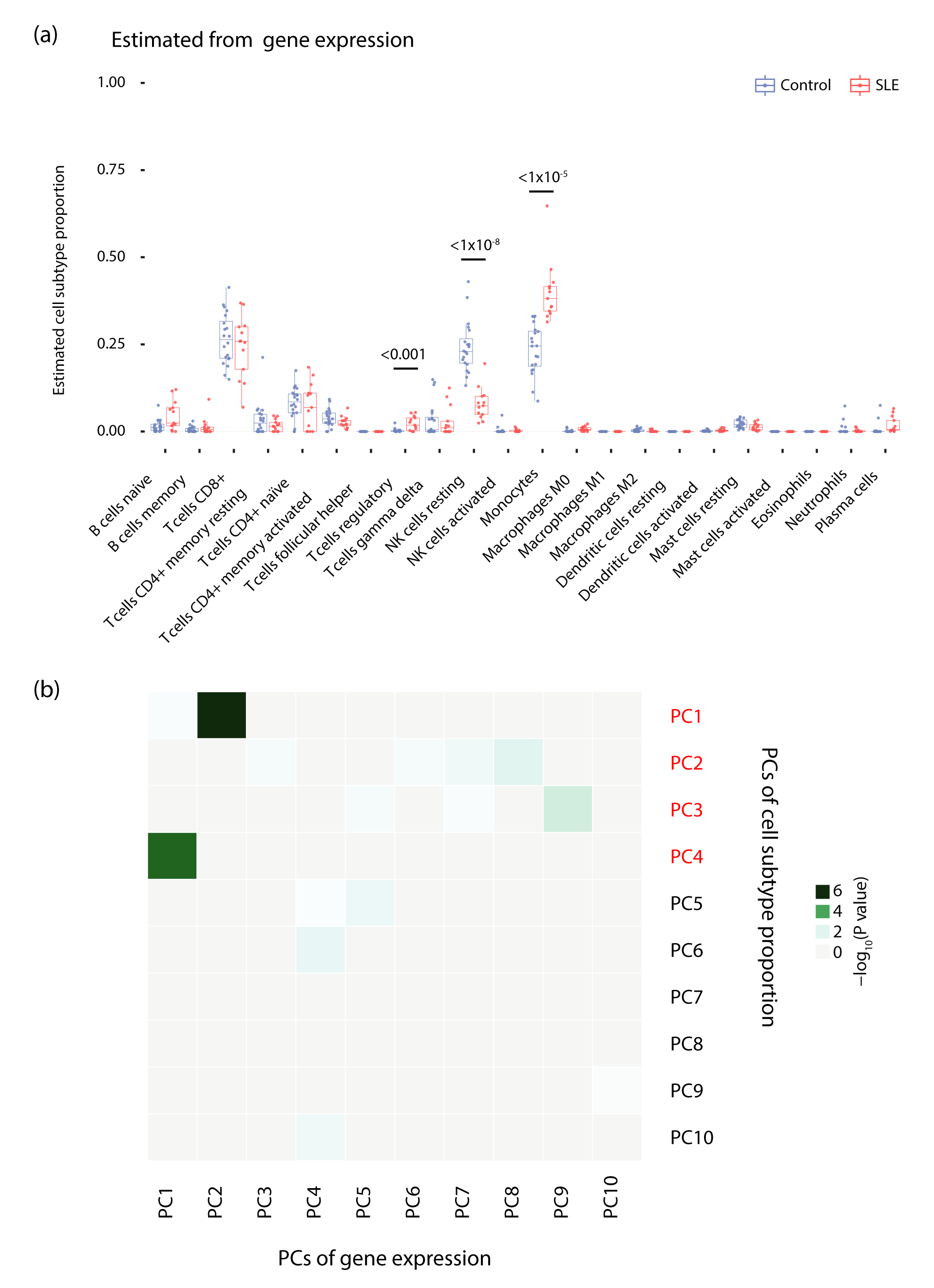

Supplement: S2 Fig — (a) The estimated cell subtype proportions using gene expression status. A boxplot showed that proportions of T-cells regulatory and monocytes were significantly increased and NK-cells resting was decreased in SLE patient. The significance was calculated with Student t-test. (b) Principal component analysis for gene expression showed significant association with PCs of cell subtype proportions estimated from expression data. The significance was calculated by a regression approach. These results suggest that the gene expression variations also strongly correlated to cell subtype proportion variations. We selected PCs with significant associations with expression variation (p-value <0.01) and which explain >1% of the variation of the cell subtype estimate for cell subtype proportion adjustment. The PCs selected using these criteria are shown in red. (TIF) [file pone.0215987.s002.tif]
