# Supplementary material for: Insights from deconvolution of cell subtype proportions enhance the interpretation of functional genomic data
Source: PLoS One. 2019 Apr 25;14(4):e0215987. doi: 10.1371/journal.pone.0215987 (PMC6483354; doi:10.1371/journal.pone.0215987)
Supplement: S1 File — (PDF) [file pone.0215987.s013.pdf]

Quality checks, preprocessing and estimating cell subtype proportions

1. GSE69683 (Asthma study, U- BIOPRED Cohorts)

1-1. Original study description and study design

This study is a cross-sectional gene expression study of severe asthmatics, moderate asthmatics, and healthy subjects. We downloaded processed data and phenotype data (498 individuals in total) from GSE69683 which used the Affymetrix HT HG-U133+ PM Array Plate (GPL13158) platform. We extracted gene information from GPL13158-5065 (https://www.ncbi.nlm.nih.gov/geo/query/acc.cgi?acc=GPL13158).

1-2. Selecting samples

The original study design consists with four groups (severe nonsmoking asthma (n=246), severe smoking/ex-smoking asthma (n=88), mild/moderate asthma (n=77) and healthy controls (n=87). Other known covariates statistics of data used can be find in the original article. Our goal was to test for cell subtype proportion effects, therefore we combined two severe asthma groups into one group and compared to healthy controls, mimicking the comparison by the authors of the original study [1].

At this stage, we have 238 females (34 healthy and 204 severe asthma asthma) and 183 males (53 healthy and 130 severe asthma asthma) samples.

1-3. Quality checks (QCs)

1-3-1. Testing gene expression status and sample identity matching

We tested gene expression profile on chromosome Y and classified sample group into two groups to check the sample mismatch. We did not observe any discordance between reported and expected sex groups.

1-3-2. Intensity distribution

We also tested the signal intensity distributions of the samples. All samples have similar intensity distributions.

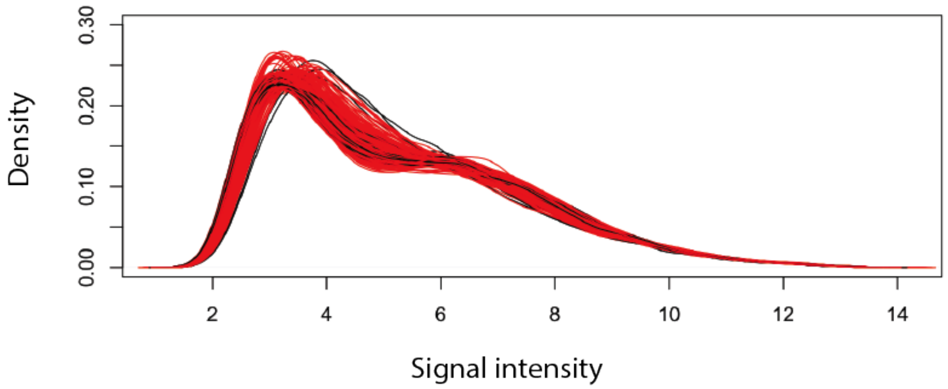

The x-axis indicates the detected gene expression intensity and the y-axis indicates the density of the probe. We colored as red is severe asthma sample, and black is healthy control.

1-3-3. Hierarchical clustering analysis

We also assessed expression profiles with hierarchical clustering approach. This allows us to see if a sever batch effects or outliers are existed in the samples.

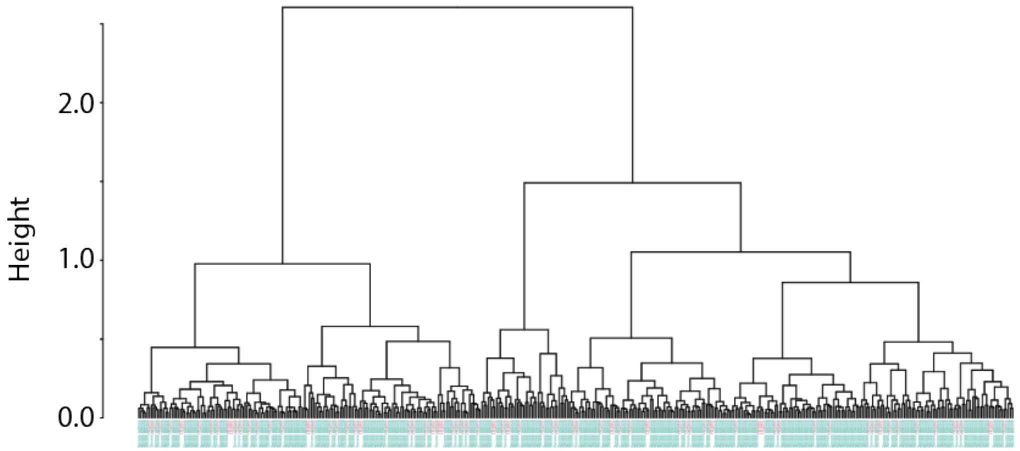

The hierarchical clustering was performed using *hclust()* function of *stats* R package using ward.D2 method. Light pink indicates healthy controls and light green indicates severe asthma patients.

Although we observed two clear branches to exist, both sex and groups were almost evenly distributed.

	Male		Female	
	Cluster 1	Cluster 2	Cluster 1	Cluster 2
Healthy	43	10	23	11
Severe asthma	74	56	123	81

1-4. Estimating cell subtype proportions with *CIBERSORT*

To estimate cell subtype proportion, we used *CIBERSORT* with the defaul LM22 (purified cell) signature genes file consisting of 547 genes which can distinguish 22 mature human hematopoietic populations. Since the LM22 signature genes file requires HUGO gene symbols as gene identifiers, we aggregated the original gene expression data by calculating median expression for each gene name. The estimated cell subtype proportion is shown in **Figure 1c.**

1-4-1. Cell subtype proportion contributions to expression data

We assessed cell subtype proportion contributions to gene expression variations. We show a heat map depicting the influences on the variability (described by principal components) of gene expression. Significant contributions of cell subtype variability are observed in principal components 1-4 of gene expression in particular. Results are shown in **Figure 1a.**

1-4-2. Testing contributions of known covariates to cell subtype proportion variations

We tested the contributions of each known covariate with a linear modeling approach. We performed principal component analysis (PCA) on cell subtype proportion variations. Following this, principal components (PCs) obtained by PCA were modeled as a linear function, such that $y \sim \alpha + \beta_{covariate} * X_{covariate} + \varepsilon$, with y indicating a PC, α indicating the intercept, $\beta_{covariate}$ and $X_{covariate}$ indicating the coefficient and the cell subtype proportion. We repeated the top 10 PCs for all known covariates. Results are shown in **Figure 1b**.

1-4-3. PCs from cell subtype proportion contributions to expression data

We tested the contribution of PCs from cell subtype proportion with a linear modeling approach. We found that PCs 1, 2, 3, 4, 5 and 9 strongly reflected the cell subtype effects on expression variation. Therefore, we used the PCs for further analysis. Results are shown in **Supplementary Figure 1.**

1-4-4. Finding differentially expressed genes (DEGs) before and after cell subtype proportion adjustment

Before the cell subtype proportion adjustment, we identified 405 DEGs (**Figure 1d**, genes listed in **S2 Table**).

We plotted the differentially expressed gene status after adjusting for the actual cell subtype proportions.

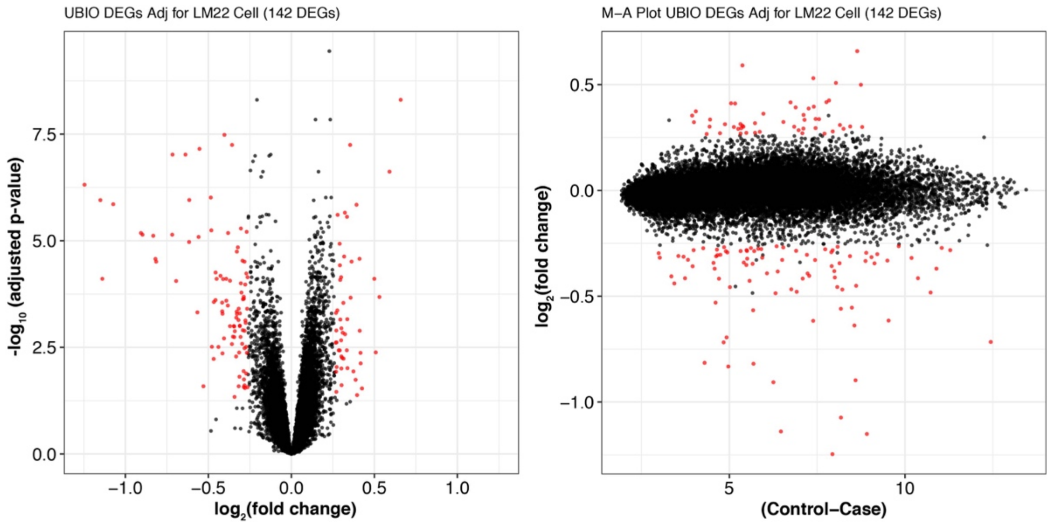

The left volcano plot showed the $\log_2$ fold change ($\log_2$ FC) with significance and the right MA-plot showed with signal intensity. The DEGs were indicated as red dots. After adjusting for actual cell subtype proportion, only 142 genes remained defined as DEGs (genes listed in **S3 Table**).

After adjusting for PCs from cell subtype proportion (PCs with association with expression data with significance level at $p<0.01$, and PCs explain >1% of variation of LM22 cell proportions were used: PC1-PC5, PC9), we identified 338 genes as DEGs (**Figure 1d**, genes listed in **S4 Table**).

1-4-5. Characteristics of eliminated and newly identified after cell subtype proportion adjustment

We listed the eliminated and newly identified genes in **S6 Table**. Most of the genes showed cell subtype dependent expression.

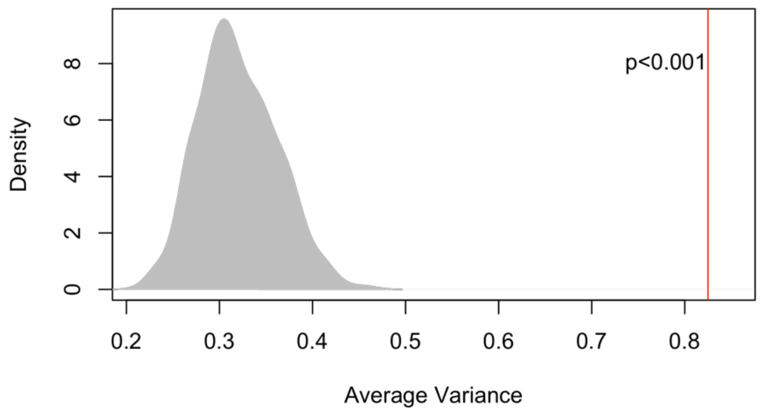

However, the gene expression variances were higher in newly identified genes. This results suggests that those newly identified genes might have gene expression alterations only in certain types of cell subtypes.

2. GSE81622/ GSE82218 (Systemic lupus erythematosus study, SLE)

2-1. Original study description and study design

This is a cross-sectional study to test the DNA methylation alterations in whole blood and transcription alterations in circulating PBMCs of 30 SLE patients, including 15 with lupus nephritis (LN) (SLE LN+) and 15 without LN (SLE LN-), and 25 normal controls (NC). We downloaded gene expression data from GSE81622 and DNA methylation data from GSE82218.

2-2. Sample selection

Comparing the estimated sex using *getSex()* function of the Bioconductor package *minfi* and the author-provided sex information, we found discrepancies for sex information in the following samples. Our result suggested that some samples were mislabeled.

Mislabeled sex samples and group

Condition	Number of samples	Mislabeled sex
NC	25 (F20, M5)	0
SLE-LN-	15 (F13, M2)	0
SLE-LN+	15(F13, M2)	4 (F2, M2)

Since all mislabeled samples were from SLE-LN+ group and the remaining number of male samples was very small, we decided to perform our analysis on the female samples from the NC and SLE-LN- groups.

2-3. Gene expression analysis

We downloaded processed data from GEO website. The authors did not provide sex and research site information.

At first, we tested the signal distribution of each individual and hierarchical clustering approach.

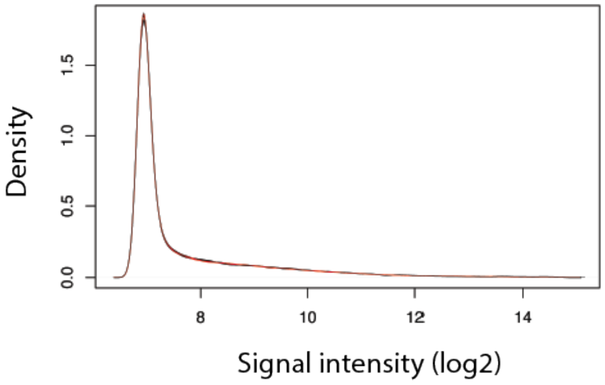

Black lines indicate the SLE cases and red lines indicate controls. We observed all samples have similar intensity distributions.

Then, we tested the dissimilarity between the samples using hierarchical clustering method.

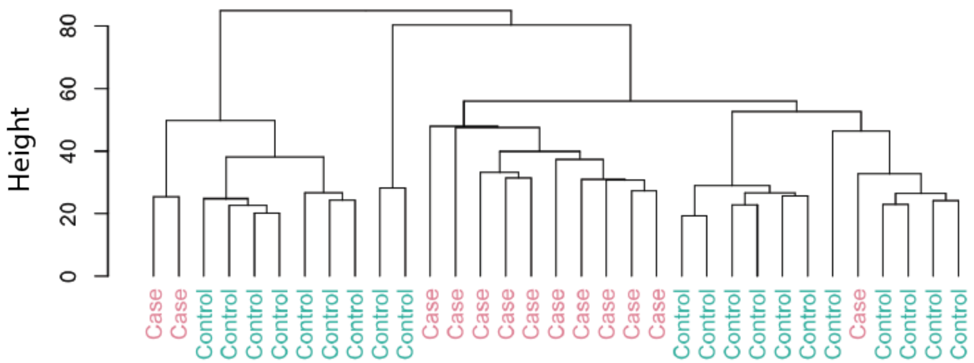

We did not observe any outliers from this analysis.

2-3-1. Testing contributions of known covariates to cell subtype proportion variations

To estimate cell subtype proportion, we used *CIBERSORT* using the default LM22 (purified cell) signature genes file. We aggregated the original gene expression data by gene name to calculate medians for each gene. Results are shown in **Supplementary Figure 2a**. We observed a significant increase of the proportion of monocytes and a decrease of resting NK cells in the patient group.

We also tested the similarity of cell subtype proportions between the individuals using Ward's minimum variance analysis [2].

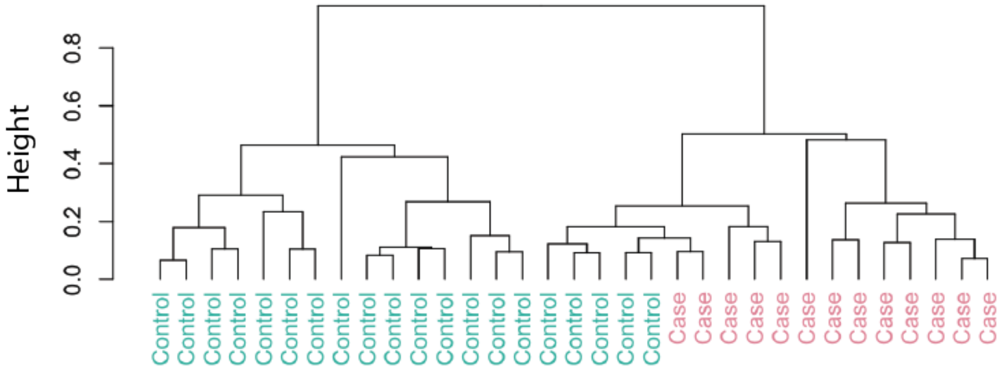

We plotted a clustering dendrogram to represent the dissimilarity between samples. We observed clear separations of cell subtype composition between case and control samples.

We performed principal component analysis (PCA) on cell subtype proportion variations and assessed the contributions of disease condition and age.

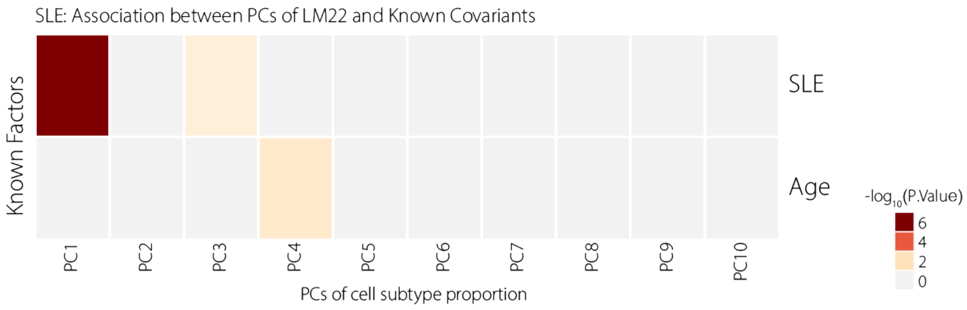

The columns show the PCs of cell subtype proportion. The disease condition was significantly associated with PC2 of cell subtype proportions.

We then tested the contribution of the known covariates and the cell subtype proportion to gene expression data variation.

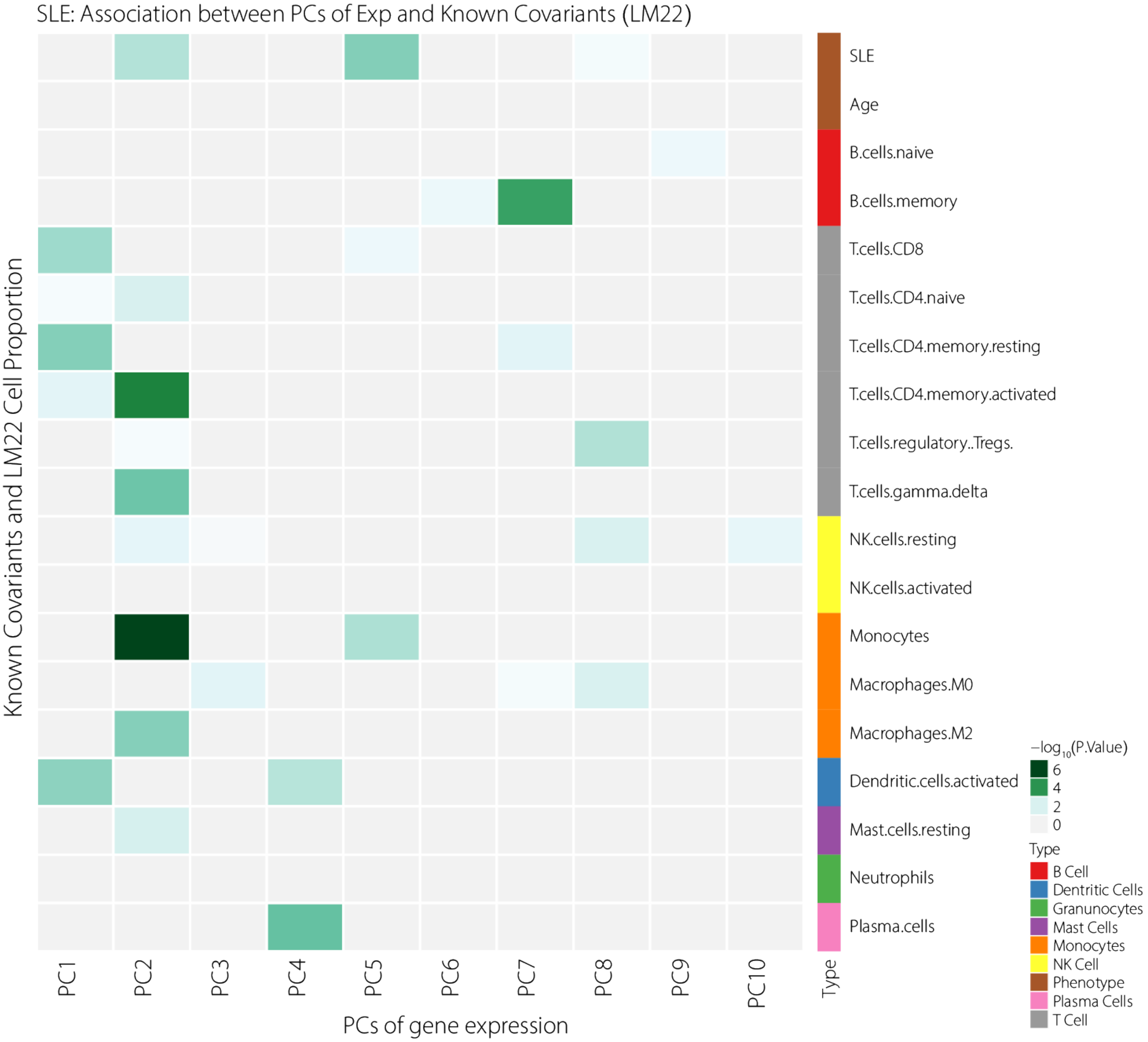

The rows show the known covariates and cell subtype proportions, and the columns show PCs of gene expression. We observed significant contributions of cell subtype proportions to PCs 1, 2, 4, 5 and 7. Also, case-control status to PCs 2 and 5. We then performed PCA on cell subtype composition to assess the significant associations between the variations of cell subtype proportions and the variation of gene expression. Results are shown in **Supplementary Figure 2b**. PCs 1 and 4 of cell subtype proportions are significantly associated with PCs 1 and 2 of gene expression variability.

2-3-2. Differentially expressed genes before and after the cell subtype proportion adjustment

We used *lmFit()* function of R pakage *limma* to identify the DEGs of before and after adjusting for cell subtype proportions between healthy control and SLE cases using significance criteria fold change > 1.5 and FDR<0.05.

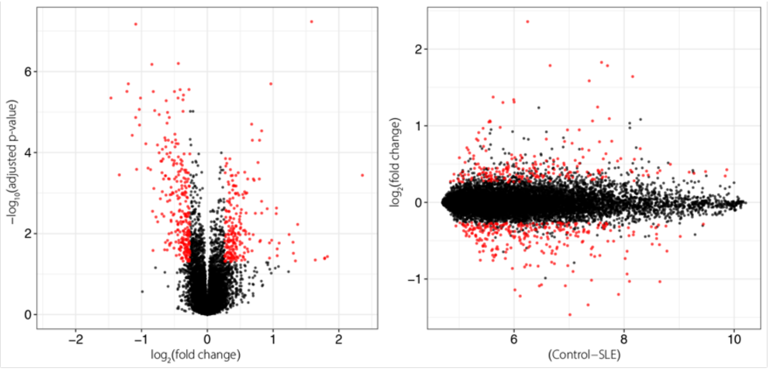

The left plot showed the $\log_2$ fold change of DEGs (red dots) with significance level and the right MA-plot showed with intensity level. Before cell subtype proportion adjustment, we found 485 DEGs between cases and controls.

Then, we performed adjustment for cell subtype proportions with actual values and PCs with association with expression data with significant level at p<0.01, and PCs explain >5% of variation of LM22 cell proportions (PC1-PC4).

Same as the plots before the adjustment, we plotted a volcano plot showing the $\log_2$ fold change of DEGs (red dots) with significance level and the MA-plot showing with intensity level.

Adjusting for actual cell subtype proportion

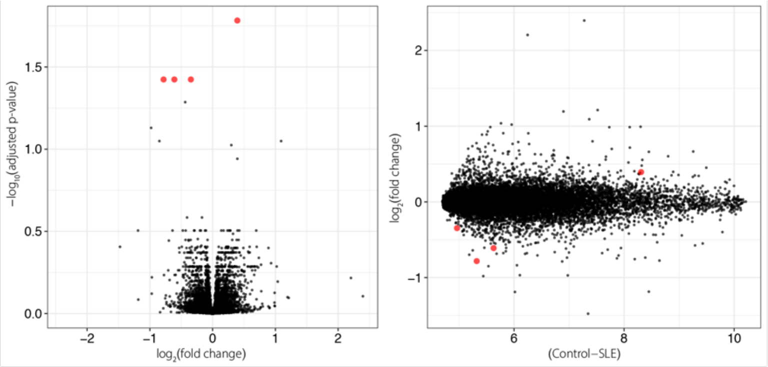

Adjusting for PC1-PC4 from cell subtype proportion

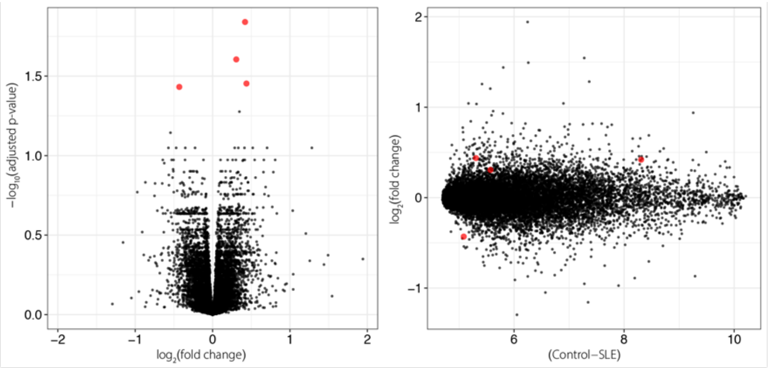

After the cell subtype proportion adjustment (both actual proportion and PCs), only four genes of the 485 DEGs without adjustment remained significant. The DEGs are listed in **S7 Table.**

This suggests that most of the DEGs identified before the cell subtype proportion were cell type-specific genes.

2-4. DNA methylation analysis

Similar to other studies, the raw .IDAT data was not available on the GEO database, so we generated the MSet data from the provided raw data table using *MethylSet()* function of the Bioconductor package *minfi*.

2-4-1. Quality check

We also used the Bioconductor package *minfi* for a quality check. We tested the average detection p-value and median intensities of the samples.

Average detection p-values:

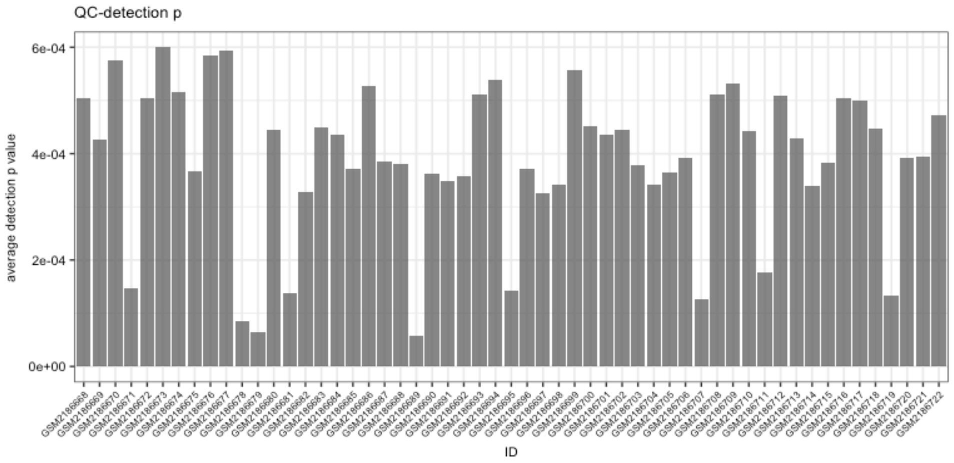

Median intensities of methylated and unmethylated probes:

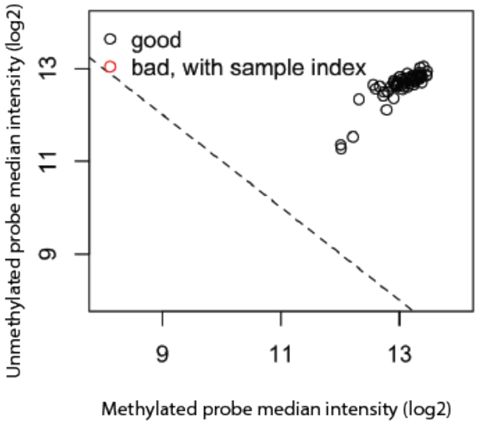

We did not observe any skewed samples in both plots. Therefore, we use all samples for the analysis.

2-4-2. Preprocessing of data

As for the other studies, we performed quantile normalization and filtering of probes.

We plotted beta-value distributions before and after the quantile normalization. Green lines indicate controls and orange lines indicate SLE patients.

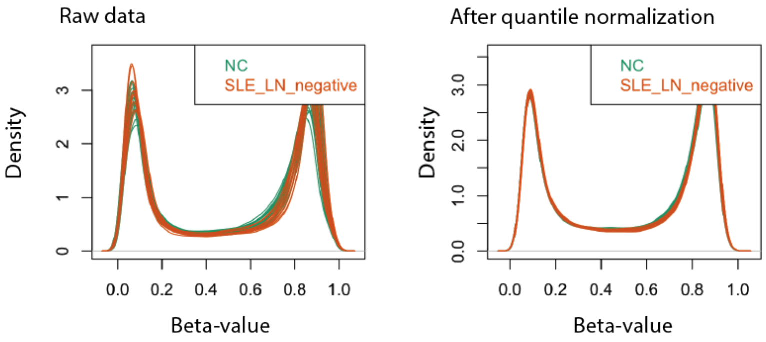

Then, we identified low-quality probes using same criteria as other studies and filtered out those probes. We plotted MDS plots before and after filtering probes (green: controls, orange: SLE patients).

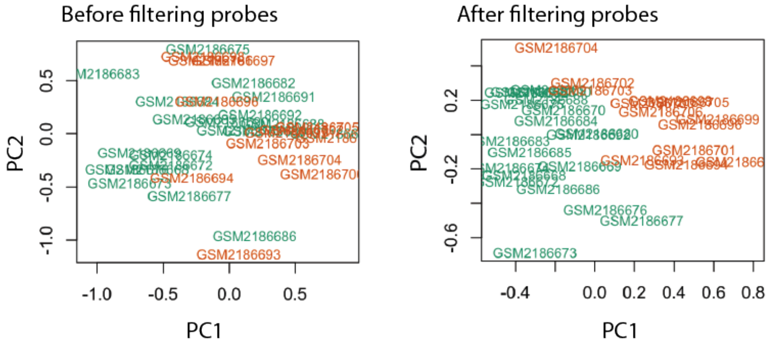

We observed disease status dependent separation after filtering low-quality probes.

2-4-3. Estimate cell subtype proportions

We used the *estimateCellCounts*() function from the Bioconductor package *minfi* to estimate cell subtype proportions. This method is a modification of the Houseman approach [3]. Because *estimateCellCounts*() only takes RGSet (raw color intensity) data as input which is not available for us, we modified the function to fit our data format. The code used this modification is available through our GitHub website (*https://github.com/GreallyLab/PBMC_Kong_2017/estimate_cell_type_function_modified.R*). Results are shown in **Figure 2a**. We observed granulocytes are significantly higher and CD4+ T cells and NK cells are significantly lower in proportions in SLE-LN- patients compared to controls.

Therefore, we performed two types of PCA. (1) PCA on the estimated cell subtype proportions, testing the association between phenotype and PCs to see if phenotype contributes to variability in cell subtype proportions, and (2) PCA on DNA methylation data, testing the association between phenotype and cell subtype proportions with PCs to see if phenotype or cell subtype proportions represent confounding factors in these DNA methylation data.

1) PCA on the estimated cell subtype proportions

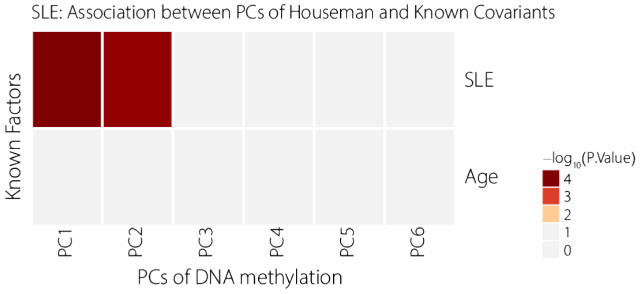

Each column indicates the PCs of cell subtype proportions. We observed that the disease condition is significantly associated with the PC1 and PC2 of cell subtype proportions.

2) PCA on DNA methylation data

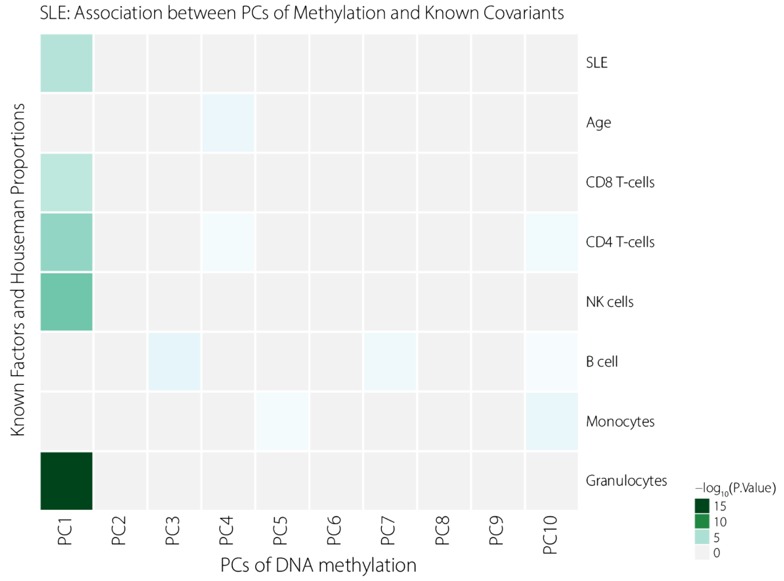

We tested the contributions of known covariates and the estimated cell subtype proportions to the PCs of DNA methylation. As we expected from the proportion differences, the proportion of T cells, NK cells and granulocytes significantly contributed to PC1 of the DNA methylation data.

We also compared the association between PCs from cell subtype proportions and PCs from DNA methylation data. Results are shown in **Figure 2b**. These results suggested that the variation of DNA methylation was mainly attributable to the cell subtype proportion differences.

To assess the degree of these contributions, we performed linear regression between PCs of DNA methylation data and the estimated cell proportions.

PC1: adjusted R^2=0.5378, p=0.0001319

	Estimate	Std.Error	t value	Pr(>|t|)
(Intercept)	-0.27021	0.01818	-14.86	3.21E-14
CD8+ T-cell	0.10116	0.01769	5.717	5.13E-06
CD4+ T-cell	0.09742	0.01964	4.96	3.74E-05
NK cells	0.09895	0.01833	5.397	1.18E-05
B-cells	0.0983	0.01867	5.265	1.67E-05
Monocytes	0.0833	0.01944	4.285	0.000222
Granulocytes	0.09579	0.01832	5.229	1.84E-05

PC2: adjusted R^2=0.949, p=2.2×10^{-16}

	Estimate	Std.Error	t value	Pr(>|t|)
(Intercept)	-0.426776	0.605664	-0.705	0.4873
CD8+ T-cell	-0.883233	0.589363	-1.499	0.146
CD4+ T-cell	0.007458	0.654257	0.011	0.991

NK cells	-0.658597	0.610601	-1.079	0.2907
B-cells	-0.172848	0.621911	-0.278	0.7833
Monocytes	1.207927	0.64748	1.866	0.0734
Granulocytes	0.720873	0.610107	1.182	0.2481

We found that 53.78% of PC 1, and 94.9% of PC2 variation can be explained by cell subtype proportions.

2-4-4. Differentially methylated probes before and after the cell subtype proportion adjustment
Before cell subtype proportion adjustment, we found 2,154 DMPs between cases and control **Figure 2c**. After adjusting for cell subtype proportions, only 40 DMPs were retained. Results are shown in **Figure 2d**. The DMPs are listed in **S8 Table.**

2-4-5. Gene ontology (GO) analysis on differentially methylated probes before and after cell subtype proportion adjustment.
We performed a GO term enrichment analysis on DMPs using the Bioconductor package *GOseq* and the obtained results were visualized with *REVIGO* [4]. After the cell subtype proportion adjustment, the type I interferon-related pathway was retained as significantly enriched. Results are shown in **Figure 2e and f**.

3. GSE40366/GSE58888 (Aging study)

3-1. Original study description and study design
This is a cross-sectional study to test the influence of aging and sex on the functional capacity of the immune system. We downloaded the data of individuals who has been analyzed in both GSE40366 (gene expression) and GSE69270 (DNA methylation).

3-2. Selecting samples and quality checks (QCs)
We selected data from the baseline for both gene expression and DNA methylation assays. In total, after normalization, 126 samples were kept for differential gene expression and differential methylation analysis.

3-3. Gene expression analysis

3-3-1. Selecting samples and QCs
We processed the gene expression profiles of 146 nonagenarians (103 females, 43 males) and 30 young controls (19-30 years of age, 21 females, 9 males). We downloaded data preprocessed by the authors. We aggregated the expression status by the gene name, calculating the mean expression level for each transcript. At this stage, we had 22,452 genes from 176 individuals. At first, we tested the signal distribution of each individual and hierarchical clustering approach.

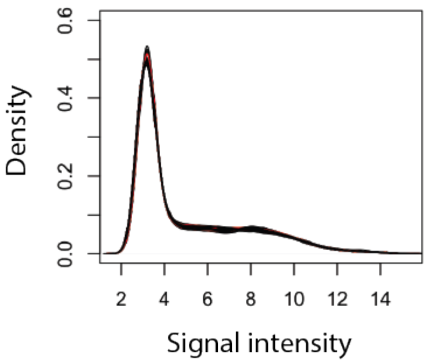

The x-axis indicates the detected gene expression intensity and the y-axis indicates the density of the probe. As we expected, the signal intensity of all samples showed similar distribution. Then we tested overall gene expression profile dissimilarity levels using hierarchical clustering approach.

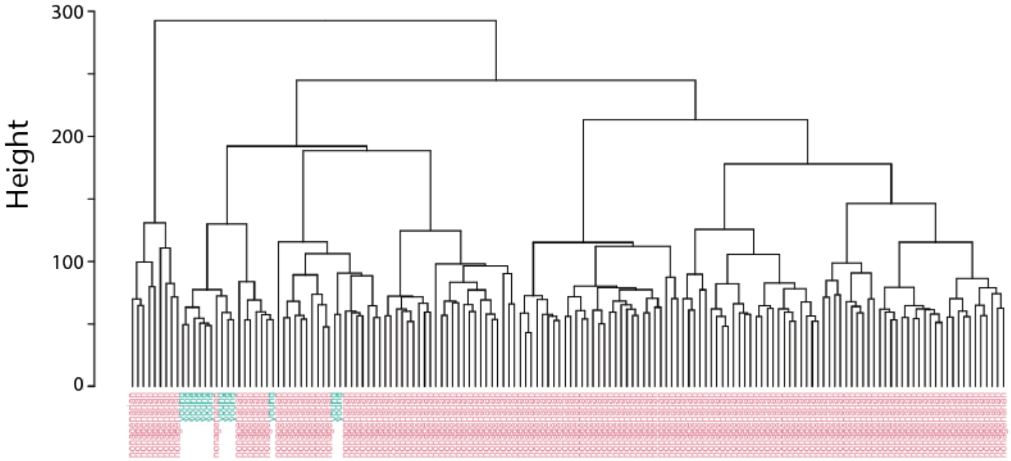

The dendrogram showed the young samples enriched in one branch. This result suggests the existence of distinct variations of gene expression profiles between nonagenarians and young controls.

3-3-2. Estimating cell subtype proportions with CIBERSORT

We estimated the cell subtype proportions of samples with *CIBERSORT* using the default LM22 reference signature gene profile.

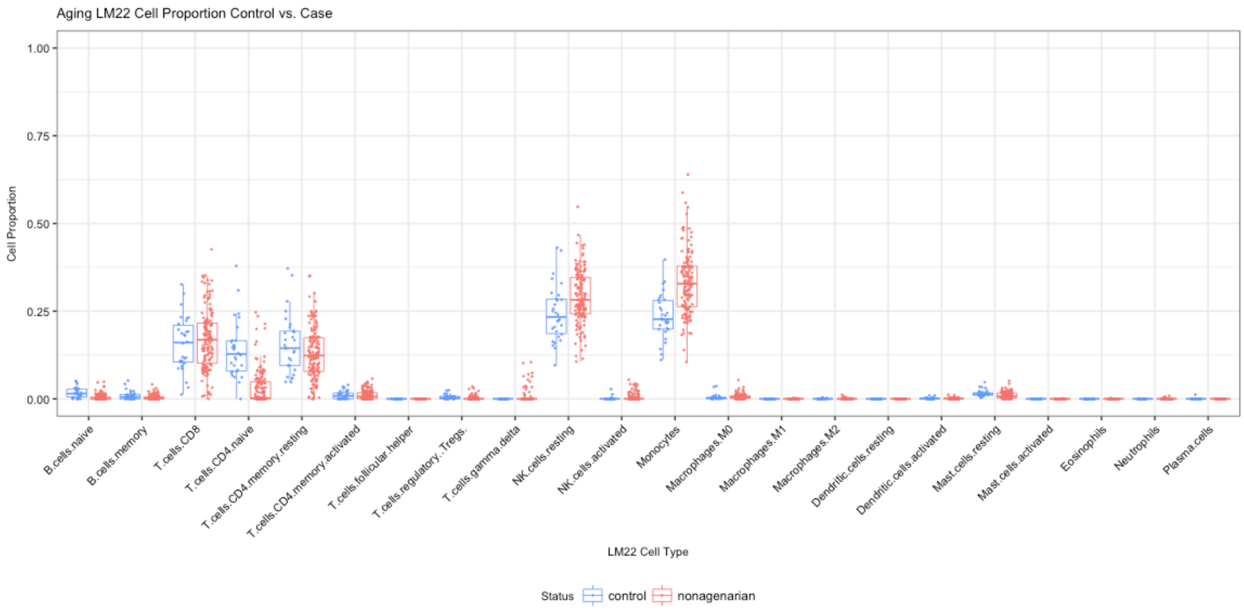

We observed significantly increased proportions of monocytes and NK resting cells and a decrease of CD4+ naïve T-cells. These findings are concordant with previous reports.

As we described above, we tested the association between cellular composition (columns) and known factors (rows) by linear regression.

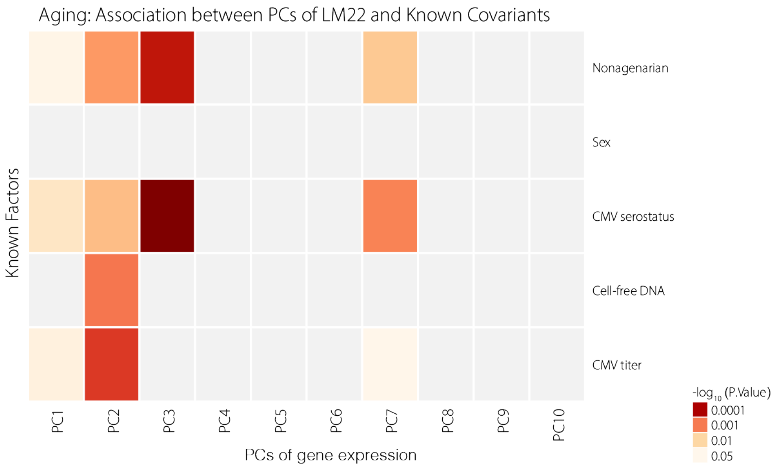

We observed that age and cytomegalovirus titer are significantly associated with the cell subtype proportions. The cytomegalovirus prevalence rate is much higher in nonagenarian groups (95.8% in nonagenarian and 63.3% in young).

We then tested the contribution of cell subtype proportions to gene expression.

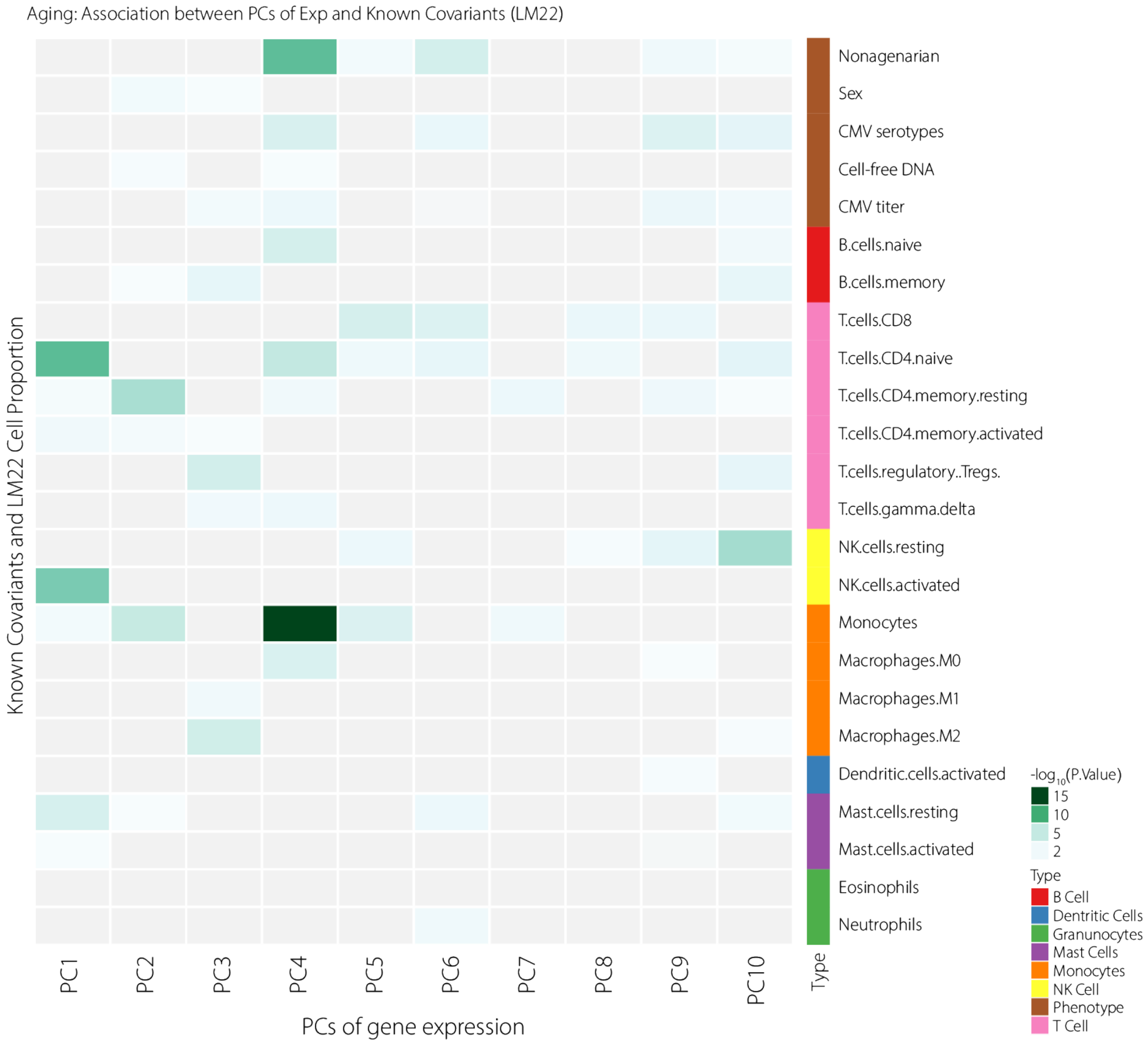

Same to the plot above, the rows indicate known covariates and cell subtype proportions, and the columns indicate the PCs of gene expression variations. As we expected from the cell proportion analysis, we observed strong contributions of proportions of CD4+ naïve T cells, activated NK cells and monocytes to the gene expression variability.

3-3-3. Finding differentially expressed genes (DEGs) before and after cell subtype proportion adjustment

We used *lmFit()* function of R package *limma* to identify the DEGs of before and after adjusting for cell subtype proportions.

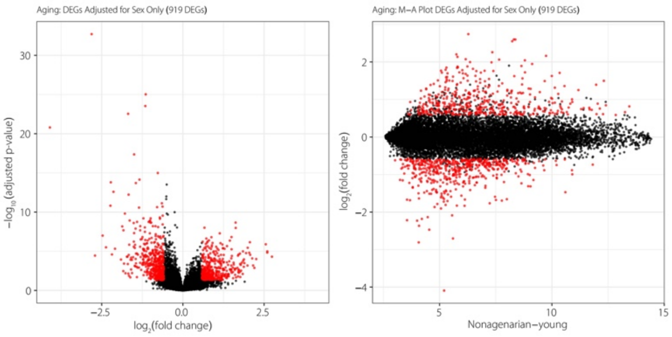

The left plot showed the log_2 fold change of DEGs (red dots) with significance level and the right MA-plot showed with intensity level. Before adjusting for cell subtype proportions, we identified 919 DEGs.

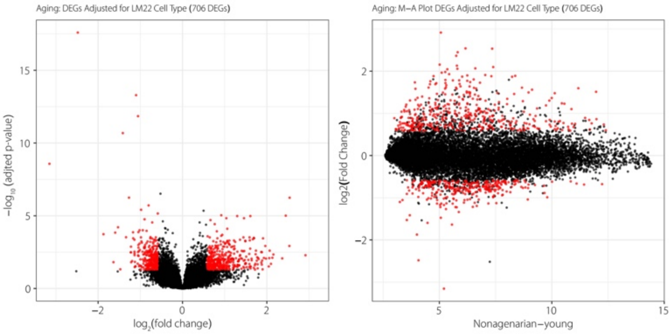

Same as the plots above, we plotted the DEGs after adjusting for actual cell subtype proportion. We identified 706 DEGs.

Then we selected PCs with association with expression data with significant level at $p<0.001$, and PCs explain >5% of variation of LM22 cell proportions (PC1-PC6) from cell subtype proportions.

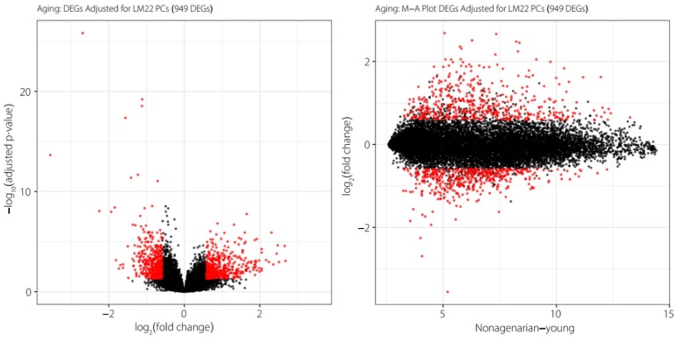

After adjusting for PCs, we identified 949 DEGs. While the number of DEGs were decreased after the cell subtype proportion adjustment using actual cell proportions, the number of DEGs were increased

after adjusting for cell proportion PCs. Therefore, adjusting for cell subtype proportion PCs helps us to detect the differences being hidden by cell subtype proportions.

3-4. DNA methylation analysis

We downloaded DNA methylation data from GSE69270 as raw beta and M values and preprocessed data using the Bioconductor package *minfi*.

3-4-1. Quality checks of data

The quality checks of data were performed using the Bioconductor package *minfi* as instructed.

To see if we have the extreme outlier of samples, we plotted the average detected p-values in the samples.

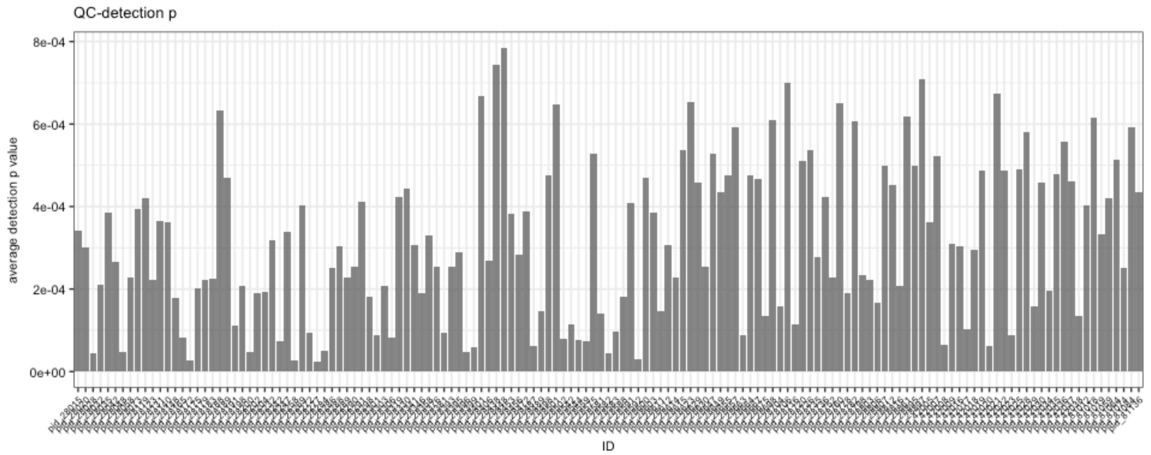

No sample has an unusually high detection P value (all<0.001). Therefore, we used all samples for the next step.

Next, we tested the distributions of medians of unmethylated and methylated probe intensities.

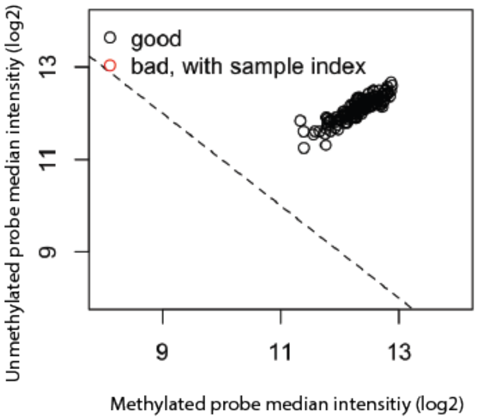

Again, we observed similar median intensities in all samples. No sample was eliminated from the analysis at this step.

3-4-2. Preprocessing of data

We performed quantile normalization using the *processQuantile* function to normalize the data, as instructed by the Bioconductor package *minfi*.

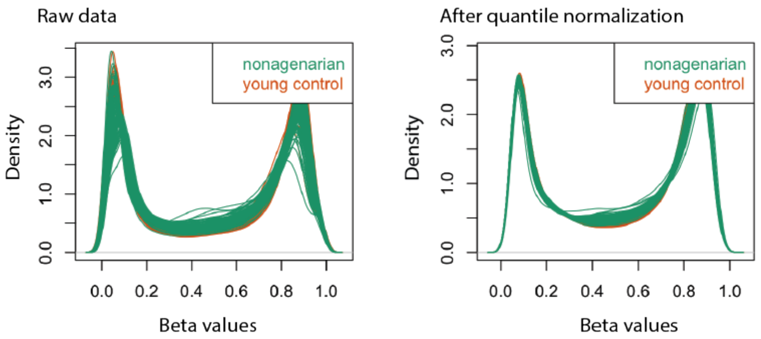

In this figure, we showed the beta-value distributions before and after the normalization. We used the quantile normalized beta-values for the analysis.

As we expected, we observed that sex clearly separated the samples in a Multi-Dimensional Scaling (MDS) plot.

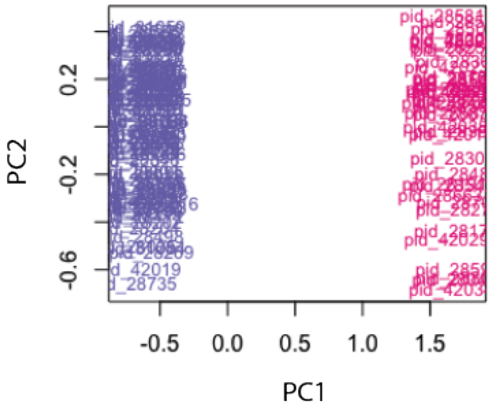

In addition, principal component analysis (PCA) showed one outlier. We decided to remove this sample from the analysis and eliminate probes on sex chromosomes.

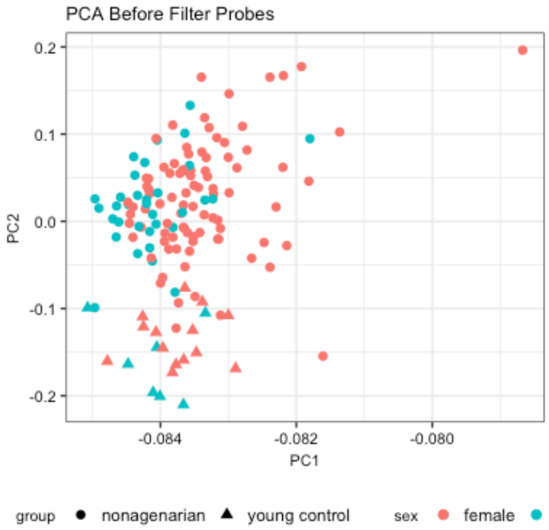

We also filtered out poorly performed probes to get more reliable downstream analysis, removing probes with the following criteria using the Bioconductor package *minfi*:

1. Probes with low detection p value (3,980 probes) using *detection()* with p>0.01 cut off
2. Probes overlapping with SNPs identified by *dropLociWithSnps()* function (16,155 probes)
3. Probes that are cross-reactive (29,234 probes) [5]
4. Probes around 1000G SNPs (1% minor allele frequency) (124,714 probes)

After filtering these probes, we performed MDS again to test the existence of other confounders.

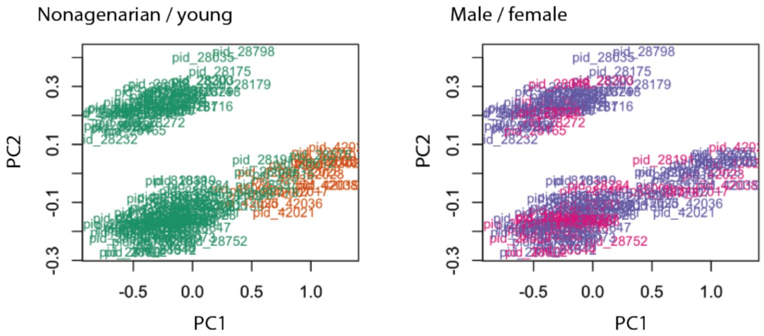

In the left panel, we colored nonagenarian samples in green and young samples in orange, and same as above we colored male samples in purple and female samples in magenta in the right panel. We observed that sex is not separated on PC1 and PC2 in the data anymore. However, PC2 in MDS clearly divided the data into two groups. According to the matrix file data supplied with the original article, the authors processed samples at two different times (6 months apart).

Therefore, we corrected for batch effect using *ComBat*() from the *sva* package. After the *ComBat* batch adjustment, we did not observe the clear separation on MDS plots.

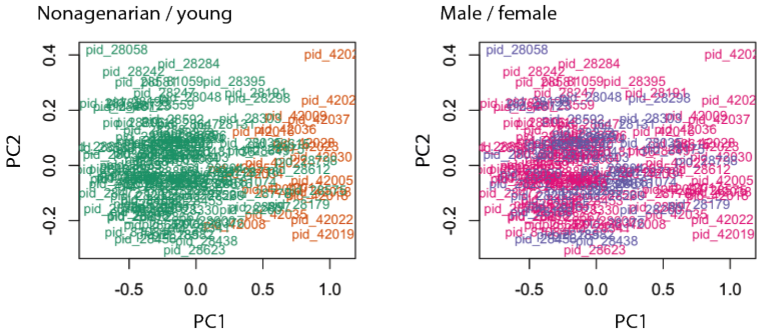

In the left panel, we colored nonagenarian samples in green and young samples in orange, and we colored male samples in purple and female samples in magenta in the right panel.

3-4-3. Estimating cell subtype proportions

We calculated batch corrected M and beta values for downstream analysis. Since the *estimateCellCounts*() function from the Bioconductor package *minfi* takes RGSet (raw color intensity) as input, we modified the function to fit our data format. The modified code is available through our GitHub repository (https://github.com/GreallyLab/PBMC_Kong_2017).

At first, we plotted the cell subtype proportion estimates by batches. We observed that both batches have similar cell subtype proportions, which suggest we successfully corrected batch effect within the DNA methylation data.

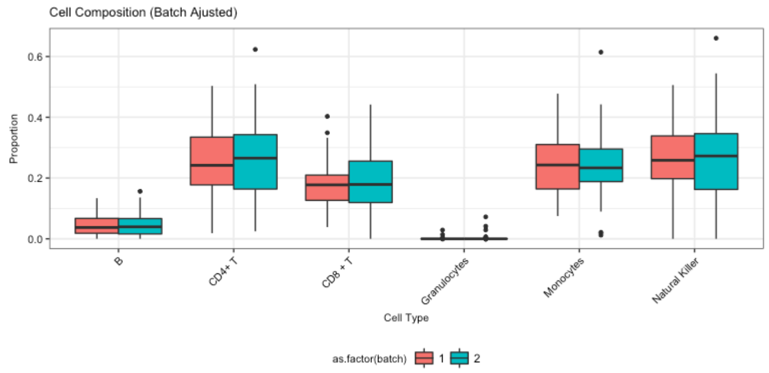

We then plotted the cell subtype proportion by groups. Concordantly with the gene expression data, we observed the proportions of natural killer (NK) cells and monocytes were higher and the proportion of CD4+T cells was lower in the nonagenarian group.

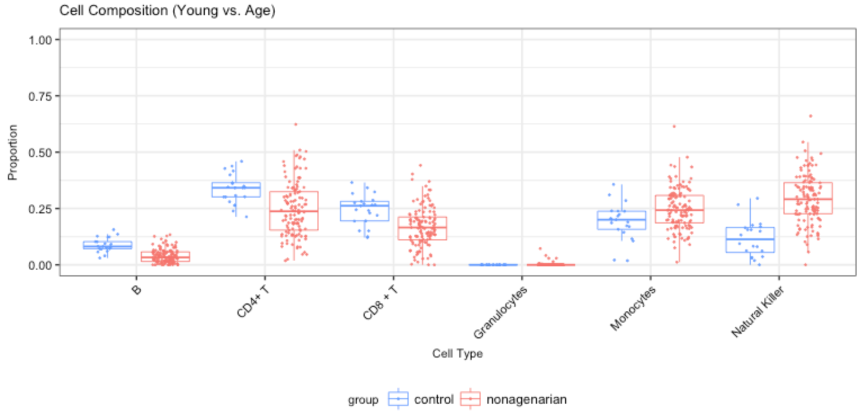

3-4-4. Finding differentially methylated probes before and after cell subtype proportion adjustment

We performed differential DNA methylation analysis using the R package *limma* to detect DMCs (differentially methylated cytosines) between young and nonagenarians using significance criteria delta-beta > 10% and FDR<0.05. Before adjusting for cell subtype proportions, we identified 12,309 differentially methylated probes (DMPs).

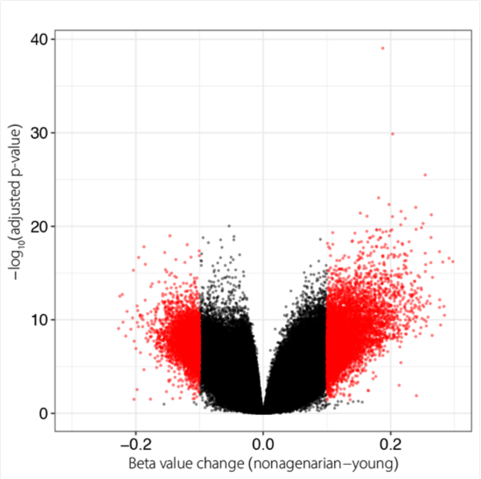

The x-axis indicates the beta value changes (nonagenarians – young) and the y-axis indicate $-\log_{10}$ (FDR-adjusted p-values). Red dots represent significant DMPs.

After adjusting for estimated cell subtype proportions, we identified 757 DMPs.

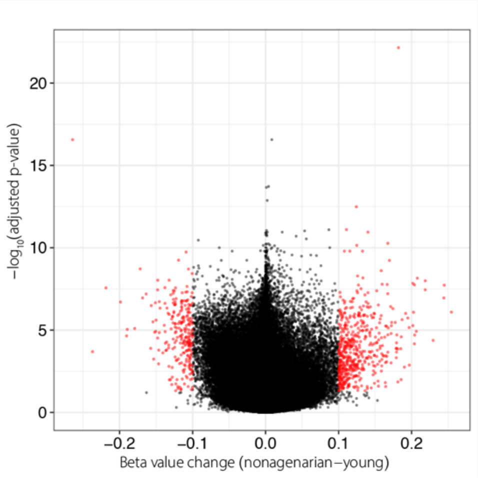

The x-axis indicates the beta value changes (nonagenarians – young) and the y-axis indicate $-\log_{10}$ (FDR-adjusted p-values). Red dots represent significant DMPs.

After adjusting for PCs (PC1, PC3 and PC4) from cell subtype proportion estimates, we identified 8,703 DMPs.

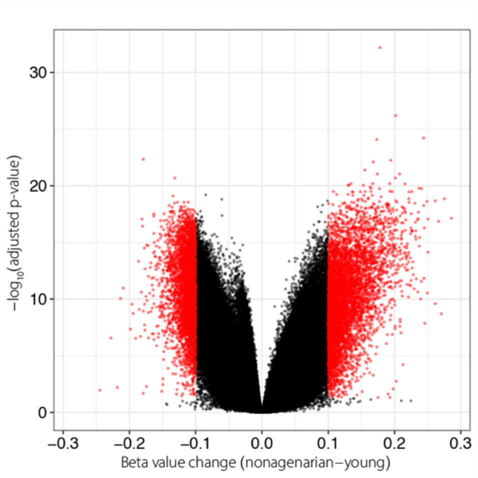

The x-axis indicates the beta value changes (nonagenarians – young) and the y-axis indicate $-\log_{10}$ (FDR-adjusted p-values). Red dots represent significant DMPs.

References

1. Bigler J, Boedigheimer M, Schofield JPR, Skipp PJ, Corfield J, et al. (2017) A Severe Asthma Disease Signature from Gene Expression Profiling of Peripheral Blood from U-BIOPRED Cohorts. Am J Respir Crit Care Med 195: 1311–1320. doi:10.1164/rccm.201604-0866OC.
2. Murtagh F, Legendre P (2014) Ward's Hierarchical Agglomerative Clustering Method: Which Algorithms Implement Ward's Criterion? J of Classification 31: 274–295. doi:10.1007/s00357-014-9161-z.
3. Houseman EA, Accomando WP, Koestler DC, Christensen BC, Marsit CJ, et al. (2012) DNA

methylation arrays as surrogate measures of cell mixture distribution. BMC Bioinformatics 13: 86. doi:10.1186/1471-2105-13-86.

4. Supek F, Bošnjak M, Škunca N, Šmuc T (2011) REVIGO summarizes and visualizes long lists of gene ontology terms. PLoS One 6: e21800. doi:10.1371/journal.pone.0021800.
5. Chen Y, Lemire M, Choufani S, Butcher DT, Grafodatskaya D, et al. (2013) Discovery of cross-reactive probes and polymorphic CpGs in the Illumina Infinium HumanMethylation450 microarray. Epigenetics 8: 203–209. doi:10.4161/epi.23470.
