# Supplementary material for: Insights from deconvolution of cell subtype proportions enhance the interpretation of functional genomic data
Source: PLoS One. 2019 Apr 25;14(4):e0215987. doi: 10.1371/journal.pone.0215987 (PMC6483354; doi:10.1371/journal.pone.0215987)
Supplement: S2 File — (PDF) [file pone.0215987.s014.pdf]

Surrogate variable analysis (SVA)

We also tested a different adjustment method, surrogate variable analysis (SVA), which adjusts for unknown covariates as a reference-free approach. Surrogate variables (SVs) are covariates constructed directly from high-dimensional data. SVA seeks to estimate the remaining unobserved factors including cell subtype proportion variations.

We used *lmFit* function of R package *limma* to identify DMPs after adjusting for SVs. We only identified 1778 DMPs. This was a much smaller number than from using the other reference-based approaches.

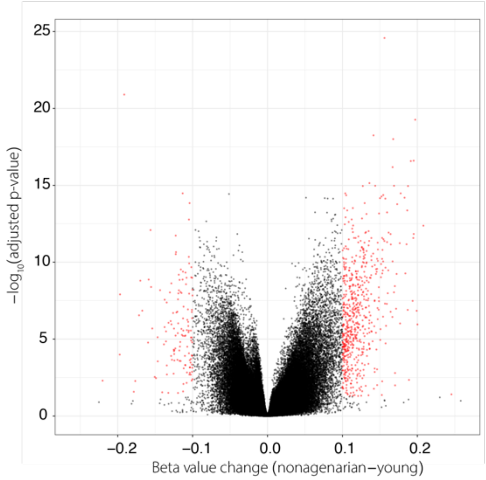

We tested the correlations of each SV to known covariates as well as cell subtype proportions. Although SVs were correlated with cell subtype proportions, SVs were also strongly and significantly associated with the phenotypic status (young/nonagenarians) in unadjusted data (**Figure 3a**), after removal of batch effect (**Figure 3b**) as well as after removal of batch and cell subtype proportion effects (**Figure 3c**). These results suggest that the SVA approach has the potential to mask some of the genuinely phenotype-associated effects.

We also performed similar analysis on gene expression analysis and observed similar trends to DNA methylation analysis.

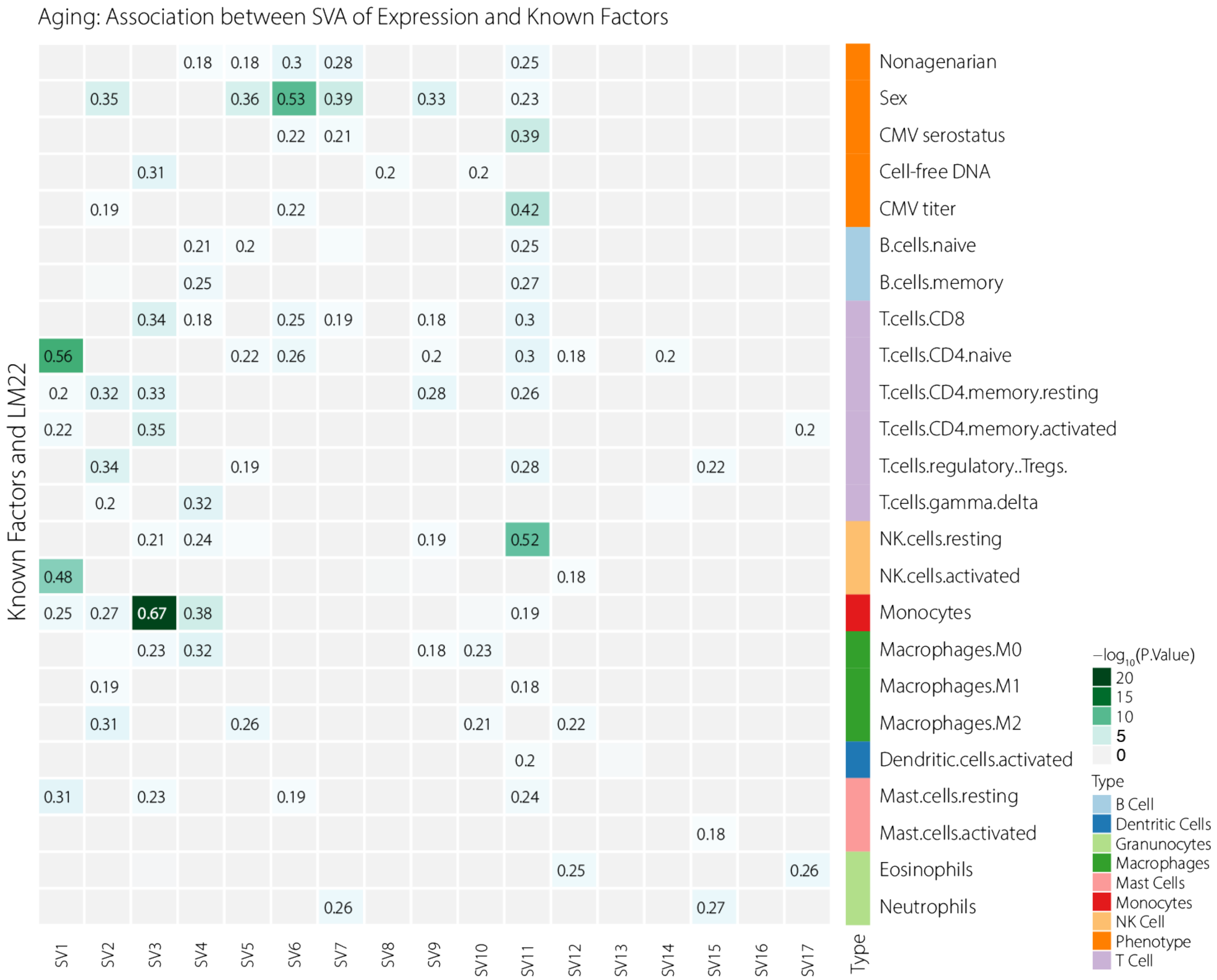

The rows show the known covariates and cell subtype proportions (LM22), and each column represents the SV of gene expression.

We also performed *lmFit()* to identify DEGs after adjustment for SVs.

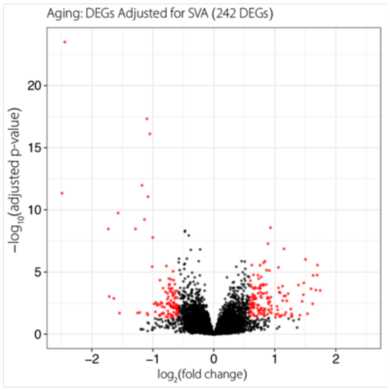

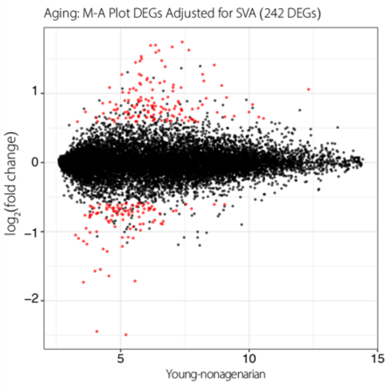

The left volcano plot showed log_2 fold changes of the DEGs (red dots) with significance and with signal intensities in the right MA-plot. After adjusting for PCs from cell subtype proportion estimates, we found 233 DEGs. After adjustment, we identified 242 DEGs.
