# Supplementary material for: Insights from deconvolution of cell subtype proportions enhance the interpretation of functional genomic data
Source: PLoS One. 2019 Apr 25;14(4):e0215987. doi: 10.1371/journal.pone.0215987 (PMC6483354; doi:10.1371/journal.pone.0215987)
Supplement: S3 File — (PDF) [file pone.0215987.s015.pdf]

Generating an e14.5 mouse kidney signature profile from single cell RNA-seq (scRNA-seq) results

Single cell RNA-seq (scRNA-seq) allows us to generate gene expression signature profiles without having prior knowledge of cell types as well as the proportion of the cell subtypes. In addition, we do not need large number of cells to distinguish each cell type. Therefore, we tested the ability of the signature profiles which we generated from the scRNA-seq data for estimating cell subtype proportions.

1. Dataset used in this study

Magella et al. reported a cross-platform single cell analysis on E14.5 mouse kidney using Drop-seq, Chromium 10x Genomics (10x genomics) and Fluidgm C1 [1]. In this analysis, we used Drop-seq and 10x Genomics data. The datasets were downloaded from the Gene Expression Omnibus (Accession GSE104396).

2. Processing scRNA-seq data

We analyzed the normalized scRNA-seq data (Drop-seq and 10x Genomics) using the Seurat R package following the supplied instructions. Drop-seq data contains 22,939 genes in 200 cells, and 10x Genomics data contains 27,998 genes in 2295 cells. We filtered genes with fewer than three cells expressing. Before merging, we eliminated cells which fewer than 1,000 genes were found to be expressed. Then, we identify the common source of variation between the two datasets using canonical correlations analysis (CCA). Before the alignment, we eliminate cells whose expression profile was not able to be explained by CCA, compared to PCA.

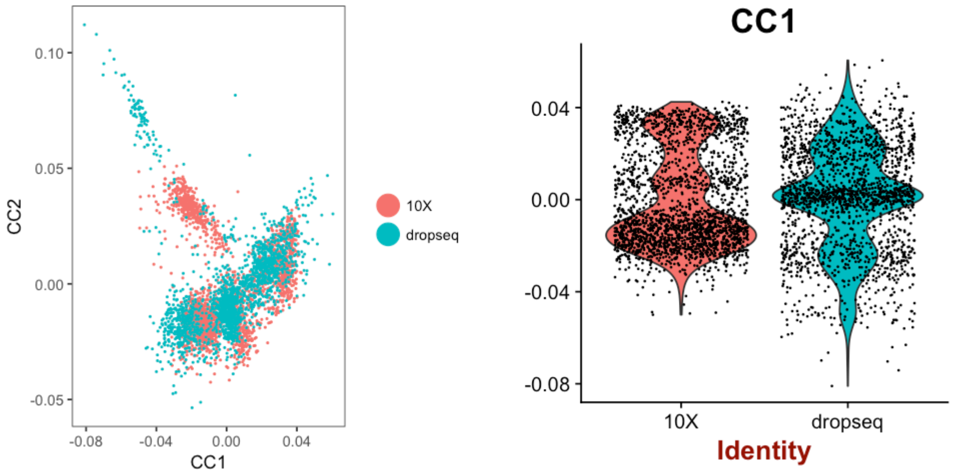

We plotted a scatterplot of CC1 and CC2 (left) and the distribution of CC1 (right). Before the alignment, the two datasets generated platform-specific cell clusters.

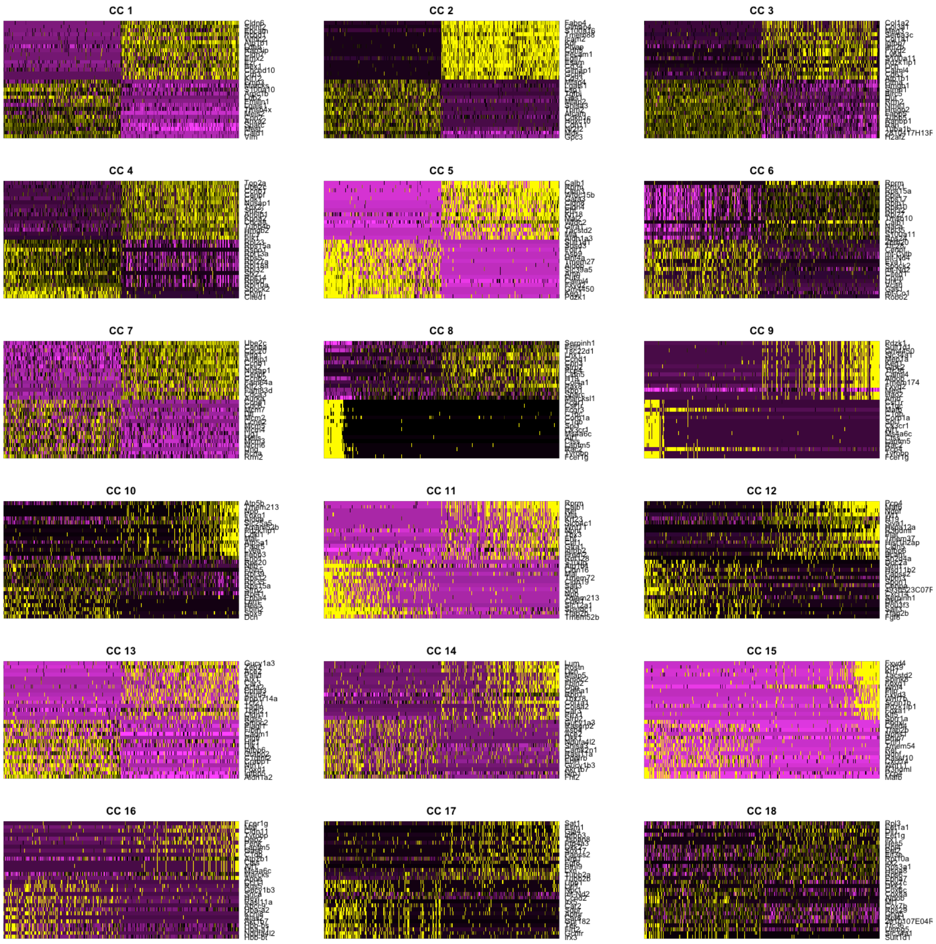

Based on the dimension heatmap analysis, we selected canonical correlations (CC) 1 to CC15 for the alignment.

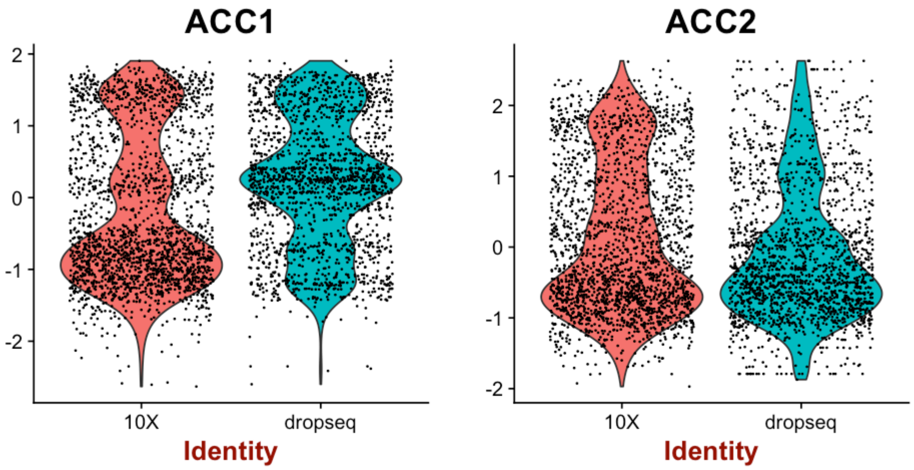

After the alignment, the platform specific distributions were eliminated in CC1 and CC2. After merging two datasets, data contains 19,592 genes in 4175 cells with a median number of detected genes per cell of 2628 (standard deviation=920.3). Then, we performed t-Distributed Stochastic Neighbor

Embedding (t-SNE) using CC1 to CC15 and find cell clusters with 1.2 resolution in Seurat FindCluster() function.

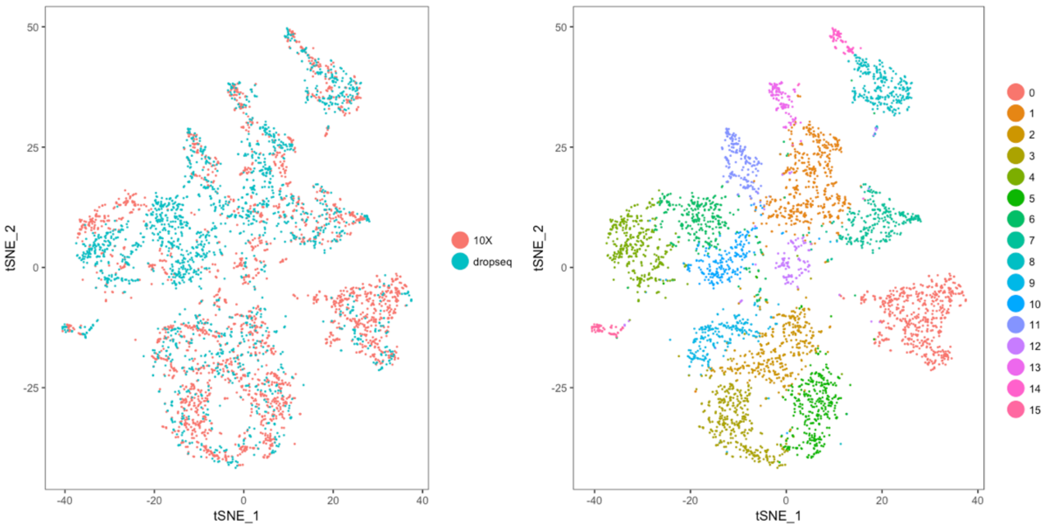

We identified cell types of each cluster based on the expression status of known cell type-specific markers (**S9 Table**).

3. Identification of cell type of each cluster and generating signature gene expression profile for each cell type

We identified 16 clusters in total, corresponding to 722 signature genes with unique expression status compared to other clusters, with on average at least 1.5-fold differences between the cluster compared with other clusters, and with at least 30% of the cells in the cluster expressing the gene (**S10 Table**). We calculated the median expression levels of the signature genes in each cluster as a reference expression signature.

Reference

1. Magella B, Adam M, Potter AS, Venkatasubramanian M, Chetal K, et al. (2018) Cross-platform single cell analysis of kidney development shows stromal cells express Gdnf. Dev Biol 434: 36–47. doi:10.1016/j.ydbio.2017.11.006.
